# An analytical approach to engineer multistability in the oscillatory response of a pulse-driven ReRAM

**DOI:** 10.1038/s41598-024-55255-7

**Published:** 2024-03-07

**Authors:** Alon Ascoli, Nicolas Schmitt, Ioannis Messaris, Ahmet Samil Demirkol, John Paul Strachan, Ronald Tetzlaff, Leon Chua

**Affiliations:** 1 Present Address: Politecnico di Torino, Department of Electronics and Telecommunications, Turin, 10129 Italy; 2 Technische Universität Dresden, Institute of Circuits and Systems, Faculty of Electrical and Computer Engineering, Dresden, 01069 Germany; 3 Peter Grünberg Institute, Forschungszentrum Jülich GmbH, Jülich, Germany; 4 RWTH Aachen University, Aachen, Germany; 5 University of California Berkeley, Department of Electrical Engineering and Computer Sciences, Berkeley, CA 94720 USA

**Keywords:** ReRAM, Nonvolatility, Fading memory, Local fading memory, Multistability, Electrical and electronic engineering, Applied mathematics

## Abstract

A nonlinear system, exhibiting a unique asymptotic behaviour, while being continuously subject to a stimulus from a certain class, is said to suffer from fading memory. This interesting phenomenon was first uncovered in a non-volatile tantalum oxide-based memristor from Hewlett Packard Labs back in 2016 out of a deep numerical investigation of a predictive mathematical description, known as the Strachan model, later corroborated by experimental validation. It was then found out that fading memory is ubiquitous in non-volatile resistance switching memories. A nonlinear system may however also exhibit a local form of fading memory, in case, under an excitation from a given family, it may approach one of a number of distinct attractors, depending upon the initial condition. A recent bifurcation study of the Strachan model revealed how, under specific train stimuli, composed of two square pulses of opposite polarity per cycle, the simplest form of local fading memory affects the transient dynamics of the aforementioned Resistive Random Access Memory cell, which, would asymptotically act as a bistable oscillator. In this manuscript we propose an analytical methodology, based on the application of analysis tools from Nonlinear System Theory to the Strachan model, to craft the properties of a generalised pulse train stimulus in such a way to induce the emergence of complex local fading memory effects in the nano-device, which would consequently display an interesting tuneable multistable oscillatory response, around desired resistance states. The last part of the manuscript discusses a case study, shedding light on a potential application of the local history erase effects, induced in the device via pulse train stimulation, for compensating the unwanted yet unavoidable drifts in its resistance state under power off conditions.

## Introduction

In their non-volatile physical realisations, resistance switching memories^1^, also known as memristors, may combine a number of functionalities within a compact nanoscale physical volume, operating alternatively as sensors, data storage devices, or signal processing units. Besides supporting multiple modes of operation, similarly as the biological synapses, non-volatile memristors also enable to exploit the third dimension in integrated circuit design, being typically arranged densely on top of Complementary Metal Oxide Semiconductor (CMOS) circuitry in the form of nanoscale oxide films, filling the gaps forming at the uniformly-spaced cross-points of arrays, employing two mutually-perpendicular sets of parallel lines, sitting along vertically-displaced yet adjacent metal layers, as rows and columns, respectively. Furthermore, memristive nanodevices allow to accelerate the execution of data detection, storage, retrieval, and processing tasks, while consuming a minuscule amount of power. The disruptive nanotechnologies, which enable the fabrication of these two-terminal bio-mimetic circuit elements, may thus allow to resolve the operating speed limitations of traditional purely-CMOS computing machines, built in accordance with the von Neumann architecture, where the separation between the physical locations, where data are respectively stored and processed, inevitably causes traffic congestion along the channel, which transfers data back and forth between memory and computing unit. Thus memristors offer the opportunity of foster progress in integrated circuit design beyond the Moore era. In fact, the aggressive rate, at which the size of CMOS transistors has been progressively shrunk over the past decades, cannot be continued much longer. Firstly, the minimum feature length is approaching atomic scales. Secondly, the ultra-high density of integration in state-of-the-art process technology nodes on one hand increases the heat inside computing machines under operation to the point, which may jeopardise the life time of its constitutive components, and on the other hand induces leakage effects, whereby currents, flowing along additional transistors’ channels besides the expected ones, may prevent CMOS circuits from the successful execution of the computing tasks they were designed for. Moreover, non-volatile memristors are the most suitable device candidates for the development of miniaturised, portable, lightweight, low-power and high-speed technical systems, which implementing innovative in-memory sensing and processing paradigms, may address the stringent requirements of Internet-of-Things and Edge Computing applications.

Since memristive one-ports are inherently nonlinear, the development of a rigorous methodology to design time- and energy-efficient circuits and systems for Artificial Intelligence applications crucially requires the adoption of concepts from *Nonlinear Circuit and System Theory*^2^, which has been unfairly overlooked in the recent past, as the typical approach of electrical engineers envisages the linearisation of the model of the device under study prior to its investigation, but this may dramatically hide fundamental aspects of its dynamics. Despite significant advancements in memristor research over the past decade, a complete understanding of the nonlinear behaviour of resistance switching memories has not been gained yet. However, drawing a complete picture of their response to any input/initial condition combination of interest for a given application is a fundamental preliminary step before a conscious and systematic methodology may be set in place to leverage their peculiar dynamics for the design of integrated circuits, which may outperform state-of-the-art purely-CMOS electronic systems, while tolerating the detrimental effects of anticipated non-idealities.

Resistive Random Access Memory (ReRAM) cells constitute one of the most important class of non-volatile memristors. Especially when tantalum oxide (TaO$_\text {x}$) or hafnium oxide (HfO$_\text {x}$) is employed as switching layer, devices of this kind feature desirable properties for data storage applications, including long retention times, high endurance, large off-to-on resistance ratios, and even multi-level capability. Moreover, recent progress in material engineering has enabled to reduce significantly the variability, which affects the electrical behaviour of a stand-alone memristor during operation and prevents matched devices, subject to the very same stimulus, while embedded in identical circuits, from displaying equivalent dynamics.

The object of the research investigations, discussed in this manuscript is a Ta$_2$O$_{5-\text {x}}$ ReRAM cell manufactured at Hewlett Packard (HP) Labs. Past research studies have already shed light into highly-nonlinear behaviour of this nano-device, in which, due to a complex interplay between ionic and electronic transport mechanisms, the resistance switching rate may vary across a multi-decade range under relatively-small changes in input strength and/or in memory state. As demonstrated through a rigorous study^3^, complementing the analysis of a predictive model^4^ from Strachan et al. (refer to section 2) with experimental tests on physical samples, the history of the nano-device under focus may be completely erased under suitable excitation. In particular, despite being able to retain the information, stored in its resistance, for a very long time under power-off conditions, an ad hoc periodic stimulus may induce the emergence of memory loss effects^5^ across its physical medium. In these circumstances, the cyclically-forced device is found to feature one and only one oscillatory behaviour, irrespective of the initial condition, after transients decay to zero. This counterintuitive phenomenon, known as *fading memory*^6^ in Nonlinear System Theory, was later observed in various other non-volatile resistance switching memories^7^. Very recently Pershin and Slipko pursued a rigorous bifurcation study^8^ of the aforementioned differential algebraic equation (DAE) set^4^ from Strachan et al. to reveal the emergence of *bistability* in the oscillatory response of the Ta$_2$O$_{5-\text {x}}$ nano-device, under the zooming lens in this manuscript, to specific two-pulse-per-cycle square wave stimuli with zero or non-zero time average and 50% duty cycle. In this regard, it is important to point out that a dynamical system, which, subject to a certain stimulus, displays either of two admissible behaviours, depending upon the initial condition, after transients vanish, is said to be subject to the simplest form of *local fading memory*^9,10^. More recently, bistable oscillations have been observed also in the memory state of another ReRAM cell, manufactured at Forschungszentrum Jülich, upon its stimulation via an ad hoc periodic stimulus, composed of a couple of triangular pulses of opposite polarity per cycle^11^. After revisiting powerful theoretical concepts, necessary for investigating the response of the ReRAM cell to periodic train stimuli, and inspired to the *time-averaging method* (consult section “The time average state dynamic route technique”) and to the *Poincaré map technique* (see section “The state change per cycle map tool”)^12^ from Nonlinear Dynamics Theory, respectively, the oscillatory response of the ReRAM cell to basic two-pulse-per-cycle square wave stimuli with non-zero time average and 50% duty cycle is thoroughly explored by means of an in-depth numerical study in section 4. Taking inspiration from the bifurcation analysis^8^ from Pershin and Slipko, the core part of this paper investigates the response of the Ta$_2$O$_{5-\text {x}}$ ReRAM cell from HP Labs to a class of generalised rectangular pulse train voltage stimuli, each of which is composed of a tuneable number $P\in \mathbb {N}_{>0}$ of positive pulses and one negative pulse per cycle, as defined in section “Adaptation of the TA-SE to a generalised pulse train stimulus”. Importantly, we propose a systematic methodology, which, exploiting the geometrical properties of the dynamic routes of the device memory state under SET resistance switching transitions (refer to section “Extraction of key geometrical features from a Gaussian bell-shaped SET state evolution function”), employs analytical formulas and solutions to a linear system of equations for determining the heights and widths of the *P* positive input pulses, as well as the width of the only negative input pulse, whose height is preliminarily prescribed, so as to endow the ReRAM cell with *P* stable oscillatory operating modes around prescribed resistance levels at steady state (consult section “A systematic methodology to craft the pulse stimulus for enabling the ReRAM cell to support multiple oscillations around prescribed resistance levels”). The proposed methodology partitions the range of admissible values for the memory state of the ReRAM cell into *P* regions, referred to as *basins of attraction*. Each of these regions contains all the initial conditions, evolving asymptotically toward an oscillatory solution, featuring as mean value a specific user-definable stable equilibrium for the ordinary differential equation (ODE), which governs the evolution of the time average of the memory state of the nanodevice under the prescribed periodic excitation. Through the proposed system-theoretic methodology the parameters of the generalised pulse train stimulus may be massaged in such a way to induce monostability or different forms of multistability (see section “Application of the theory to endow the ReRAM cell with three, four, or five oscillatory behaviours”) in the device oscillatory behaviour upon request. The capability of the ReRAM cell to act as a monostable or multistable oscillator under suitable periodic pulse train stimulation may be leveraged to develop novel forms of data detection and computation in memory for artificial intelligence applications in the years to come. For example, as revealed in section “Compensating for the drift in the resistance of crosspoint devices under power off conditions”, the regular application of a suitable generalised pulse train voltage signal across each crosspoint nanodevice may allow to correct an unwanted drift in its resistance state under power off conditions. Finally, the conclusions, summarising the most significant results of this research study, are drafted in section “Conclusions”.

## Memristor Model

The Strachan model^4^ falls in the class of first-order extended voltage-controlled memristors^13^, defined via the DAE set^14^

1 $$\begin{aligned} \dot{x}= & {} g(x,v), \text {and} \end{aligned}$$

2 $$\begin{aligned} {i}&{=}&{G(x,v) \cdot v,} \end{aligned}$$

where the ODE (1), referred to as *state equation*, dictates the rate of change of the memory state *x* of the one-port, as an input voltage signal *v* is let fall between its terminals. The algebraic constraint (2), known as *state- and input-dependent Ohm law*, defines how state and voltage affect the flow of the output current signal *i* through the device stack. In (1) ((2)) *g*(*x*, *v*) (*G*(*x*, *v*)) represents the state evolution (memductance) function. Let us assume *x* to be constrained to lie at all times within a closed set $\mathscr {D} \triangleq [x_{\text {L}},x_{\text {U}}]$. In the Strachan model the state evolution function reads as

3 $$\begin{aligned} g(x,v)= & {} g_{\text {SET}}(x,v) \cdot \text {step}(v)+g_{\text {RESET}}(x,v) \cdot \text {step}(-v), \end{aligned}$$

where $\text {step}(\cdot{)}$ is the Heaviside function, while the SET $g_{\text {SET}}(x,v)$ and RESET $g_{\text {RESET}}(x,v)$ components of *g*(*x*, *v*), referred to as *SET and RESET state evolution functions*, and governing the evolution of the device memory state under positive and negative input voltages, respectively, are in turn defined as

4 $$\begin{aligned}{} & {} g_{\text {SET}}(x,v)\triangleq B \cdot \sinh \left( \frac{v}{\sigma _{\text {on}}}\right) \cdot \exp \left( -\frac{x^2}{x_{\text {on}}^2}\right) \cdot \exp \left( \frac{p}{\sigma _{\text {p}}}\right) , \text {and} \end{aligned}$$

5 $$\begin{aligned}{} & {} g_{\text {RESET}}(x,v)\triangleq A \cdot \sinh \left( \frac{v}{\sigma _{\text {off}}}\right) \cdot \exp \left( -\frac{x_{\text {off}}^2}{x^2}\right) \cdot \exp \left( \frac{1}{1+\beta \cdot p}\right) , \end{aligned}$$

in which $p=i \cdot v$ denotes the power dissipated in the memristor, as a voltage signal is applied between its terminals. The formula for the memductance function in the Strachan model assumes the form

6 $$\begin{aligned} G(x,v)=G_{\text {m}} \cdot x+ a \cdot \exp \left( \,b \cdot \sqrt{|v|}\,\right) \cdot (1-x). \end{aligned}$$

For any given voltage *v*, the higher is the memory state *x*, and the larger is the memductance. In the remainder of this paper, the ReRAM cell model, consisting of the ODE (1), with state evolution function (3), where the SET and RESET components are respectively expressed by equations (4) and (5), and of the algebraic relation (2), with memductance function (6), is referred for simplicity as *Strachan DAE set*. Table 1 reports the values assigned to the parameters in Eqs. (4), (5), and (6) so as to allow the resulting model to reproduce experimental data, extracted from a Ta$_2$O$_{5-\text {x}}$ physical sample, to within a preliminarily specified degree of accuracy^3^.

**Table 1:** Strachan model parameters fitted to a Ta$_2$O$_{5-\text {x}}$ physical sample.

$A\,/{\text{s}^{-1}}$	$\sigma _{\text {off}}\,/\text{V}$	$x_{\text {off}}$	$\beta \,/{(\text{A}^{-1} \times \text{V}^{-1})}$
$10^{-10}$	$1.3\times 10^{-2}$	$4 \times 10^{-1}$	500
$B\,/{\text{s}^{-1}}$	$\sigma _{\text {on}}/\text{V}$		$x_{\text {on}}$
$1\cdot 10^{-4}$	$4.5 \times 10^{-1}$		$6 \times 10^{-2}$
$\sigma _{\text {p}}\,/{(\text{A}^{-1} \times \text{V}^{-1})}$	$G_{\text {m}}\,/{\Omega ^{-1}}$	$a\,/{\Omega ^{-1}}$	$b\,/{\text{V}^{-1/2}}$
$4 \times 10^{-5}$	$2.5 \times 10^{-2}$	$7.2 \times 10^{-6}$	4.7

The lower $x_{\text {L}}$ and upper $x_{\text {U}}$ bounds in the state existence domain $\mathscr {D}$ are respectively equal to 0 and 1.

## Theoretical tools

This section introduces the theoretical concepts applied in the research study discussed later on.

### The time average state dynamic route technique

**Figure 1:**
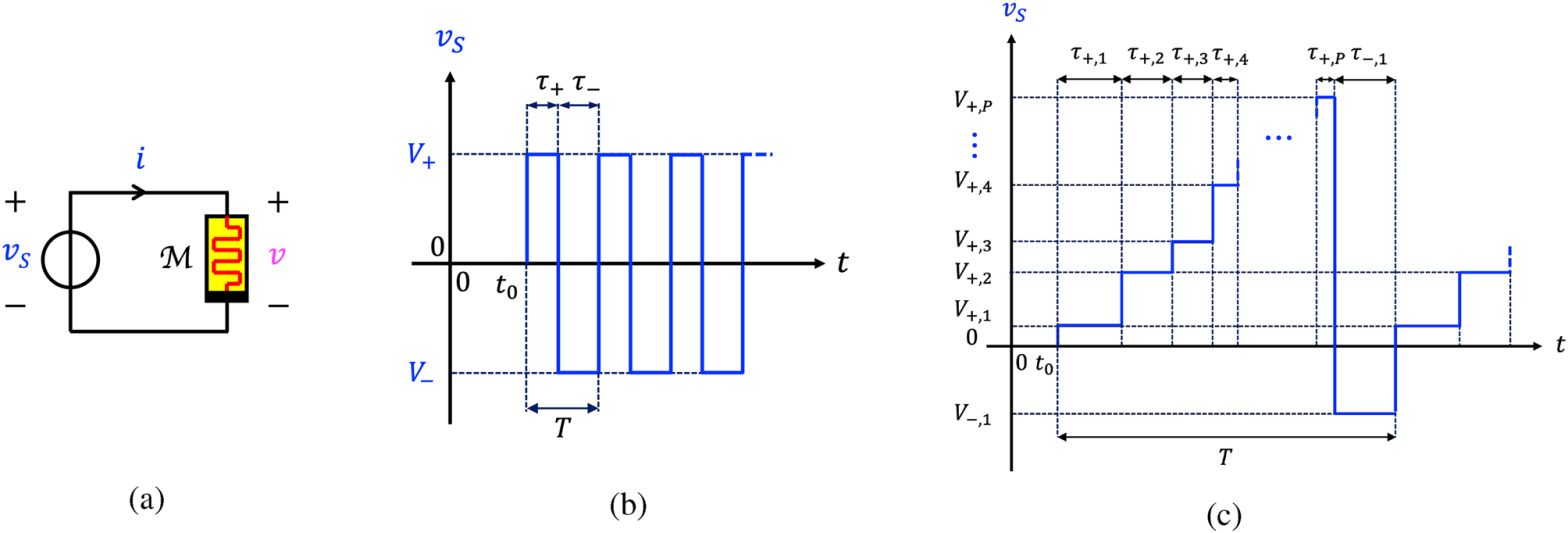
(**a**) Circuit employed to investigate the response of the Ta$_2$O$_{5-\text {x}}$ ReRAM cell^15^ to periodic square pulse-based voltage excitation signals. (**b**) Time course of a two-pulse-per-cycle train voltage stimulus. (**c**) Time waveform of a generalised pulse train voltage stimulus $v_S$, including *P* positive SET pulses and one negative RESET pulse per cycle. The RESET pulse of height $V_-$ and width $\tau _{-}$ follows the series of SET pulses. The $i$th SET pulse is $V_{+,i}$ high and $\tau _{+,i}$ wide, with $i\in \{1,\ldots ,P\}$. *The ordering of the positive pulses from the lowest to the highest in each input cycle follows the convention adopted in the systematic methodology to engineer multistability in the steady-state oscillatory response of the ReRAM cell to a generalised train stimulus* (refer to section “A systematic methodology to craft the pulse stimulus for enabling the ReRAM cell to support multiple oscillations around prescribed resistance levels”. However, this has no effect on the simulations. In fact, to facilitate their convergence, in the numerical investigations, discussed in section “Conclusions”, the SET pulses were listed from the most narrow to the most wide before being applied in this order one after the other across the device.

When a voltage signal $v_S$ falls across the ReRAM cell, as illustrated in Fig. 1a, the time average $\bar{x}$ of its memory state *x*, referred to as *time average state* for short, evolves with time according to the formula

7 $$\begin{aligned} \bar{x}(t)=\frac{1}{T} \cdot \int _{t}^{t+T} x(t') dt'. \end{aligned}$$

Taking the time derivative of both sides of Eq. (7) gives

8 $$\begin{aligned} \dot{\bar{x}} = \frac{x(t + T) - x(t)}{T}=\frac{1}{T} \cdot \int _{t'=t}^{t'=t + T} g(x(t'), v_S(t')) dt', \end{aligned}$$

where the last step follows from the integration of the state equation (1) for $v=v_S$. In principle Eq. (8) could be employed to explore the response of the device to any periodic stimulus, but then recurring to some numerical integration method would be necessary for determining its solutions.

However, as explained shortly, for stimuli composed of rectangular pulses of suitable widths and heights, the study of the behaviour of the periodically-forced device may be considerably simplified, and some analytical developments, following from a simplification of Eq. (8), are possible. Let us first consider the excitation scenario analysed in the bifurcation study from Pershin and Slipko^8^. With reference to Fig. 1b, the train voltage stimulus $v_S$, applied across the memristor, features here a first $\tau _+$-long SET pulse of positive polarity and amplitude $V_+$ and a second $\tau _-$-long RESET pulse of negative polarity and amplitude $V_-$ over each cycle of length $T=\tau _++\tau _-$. Equation (8) reduces then to

9 $$\begin{aligned} \dot{\bar{x}} =\frac{1}{T} \cdot \int _{t}^{t + \tau _+} g_{\text {SET}}(x(t'), V_+) dt'+\frac{1}{T} \cdot \int _{t+\tau _+}^{t + T} g_{\text {RESET}}(x(t'), V_-) dt'., \end{aligned}$$

**Figure 2:**
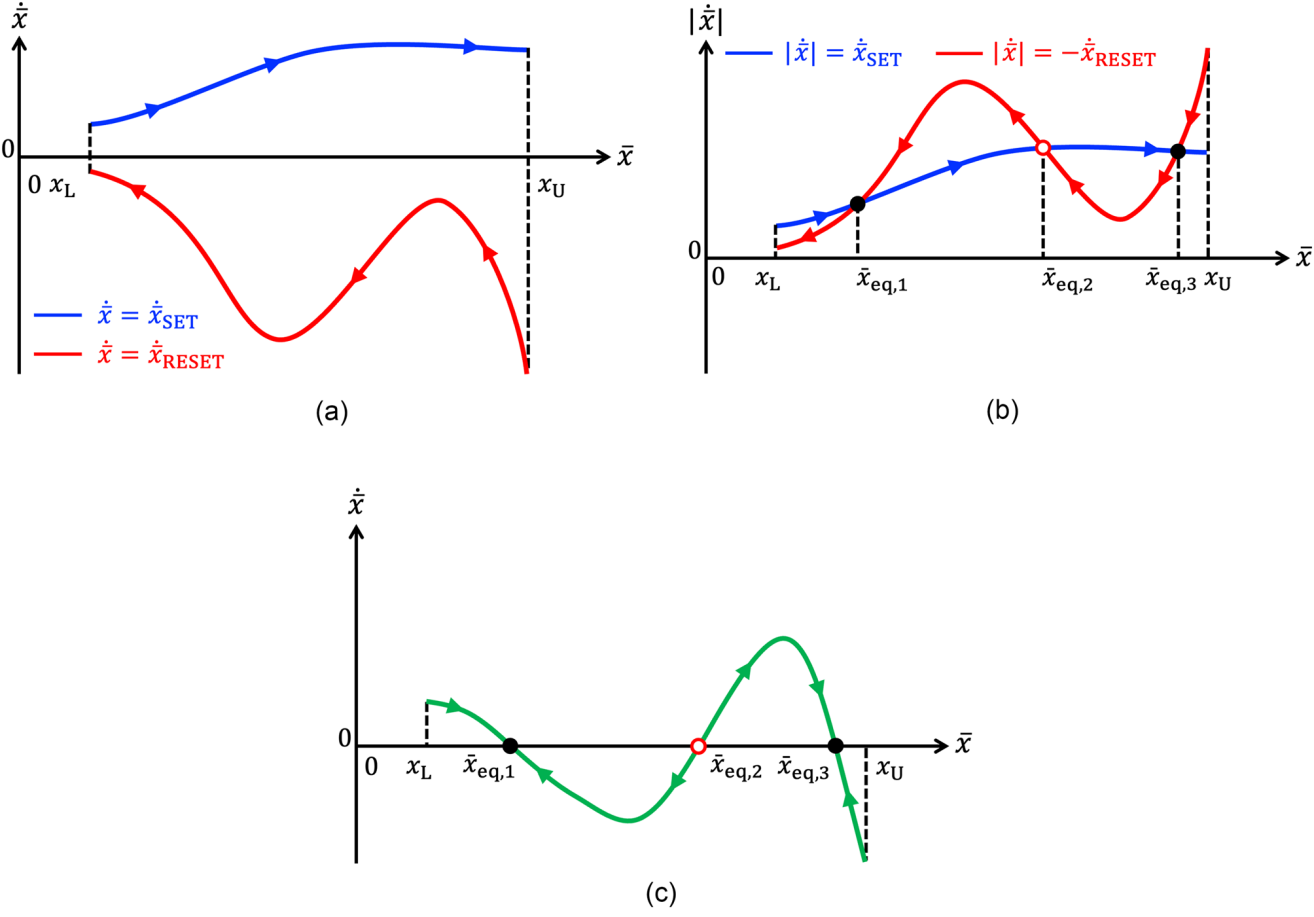
(**a**) Blue (Red) trace: SET $\dot{\bar{x}}_{SET}$ (RESET $\dot{\bar{x}}_{RESET}$) component of the TA-SE (10) of the ReRAM cell under an arbitrary pulse train stimulation. (**b**) Moduli of the SET and RESET TA-SE components. Their intersections identify the TA-SE equilibria. (**c**) TA-SDR of the ReRAM cell subject to the arbitrarily chosen pulse train stimulus. Arrows, pointing to the east (west), are superimposed along any TA-SDR branch, which visits the upper (lower) half plane, so as to indicate a progressive increase (decrease) in the time average state $\bar{x}$ when $\dot{\bar{x}}$ is positive (negative). An equilibrium for the TA-SE exists at the abscissa $\bar{x}=\bar{x}_{eq}$ of any point, at which the TA-SDR crosses the horizontal axis, as $\dot{\bar{x}}=0$ therein. The equilibrium is asymptotically stable (unstable), as indicated via a black filled (red hollow) circle, if and only if the slope $\partial \dot{\bar{x}} / \partial \bar{x}$ of the $\dot{\bar{x}}$ versus $\bar{x}$ locus is negative (positive) at its location. According to the TA-SDR analysis, the ReRAM cell is expected to act as a bistable oscillator under the given periodic excitation.

Assuming the positive (negative) SET (RESET) pulse induces a relatively small increase (decrease) in the device memory state over the first (second) $\tau _+$ ($\tau _-$)-long part of each cycle, it is possible to substitute the state *x* with its time average $\bar{x}$ in each integrand without introducing a large error in the resulting approximation. Equation (9) boils then down to

10 $$\begin{aligned} \dot{\bar{x}} \approx \dot{\bar{x}}_{\text {SET}}+\dot{\bar{x}}_{\text {RESET}}, \end{aligned}$$

where

11 $$\begin{aligned} \dot{\bar{x}}_{\text {SET}}= & {} \tilde{\tau }_{+} \cdot g_{\text {SET}}(\bar{x},V_+), \text {and} \end{aligned}$$

12 $$\begin{aligned} \dot{\bar{x}}_{\text {RESET}}= & {} \tilde{\tau }_{-} \cdot g_{\text {RESET}}(\bar{x},V_-), \end{aligned}$$

with $\tilde{\tau }_{+} \triangleq \tau _+/T$ and $\tilde{\tau }_{-} \triangleq \tau _-/T$. This ODE, referred to as *time average state equation* (TA-SE)^8^, governs the time evolution of the time average state $\bar{x}$ of the memristor when a voltage source, generating a specific square pulse train voltage stimulus $v_S$, belonging to the class illustrated in Fig. 1b, and characterised by the parameter quartet $(V_+,\tau _+,V_-,\tau _-)$, is connected between its terminals, as shown in plot (a) of the same figure. Equations (11) and (12) are respectively referred to as *SET and RESET TA-SE components*. The blue (red) trace in Fig. 2a illustrates qualitatively a $\dot{\bar{x}}_{\text {SET}}$ ($\dot{\bar{x}}_{\text {RESET}}$) versus $\bar{x}$ locus of the ReRAM cell subject to an arbitrary pulse train voltage stimulus. The SET (RESET) resistance switching process tends to increase (decrease) the time average state over each input cycle, as indicated by the arrows on the first (latter) single-valued curve. Plotting the RESET TA-SE component in modulus, as depicted in plot (b) of the same figure, allows to visualise clearly each point, at which the SET and RESET forces balance out. A point of this kind denotes an *equilibrium* $\bar{x}=\bar{x}_{eq}$ for the TA-SE, as $|\dot{\bar{x}}_{SET}|_{\bar{x}=\bar{x}_{eq}}=|\dot{\bar{x}}_{RESET}|_{\bar{x}=\bar{x}_{eq}}$ implies $\dot{\bar{x}}=0$. A TA-SE equilibrium is asymptotically stable if and only if $|\dot{\bar{x}}_{SET}|>(<)|\dot{\bar{x}}_{RESET}|$ locally to the left (right) of its location, and *unstable* otherwise. A blue filled circle (red hollow circle) is employed to indicate the location of a stable (an unstable) TA-SE equilibrium. The $\dot{\bar{x}}$ versus $\bar{x}$ locus, derivable by summing the ordinates of the vertically-aligned points, sitting along the SET and RESET traces from plot (a), for each $\bar{x}$, as dictated by Eq. (10) (refer to Fig. 2c), is the so-called *time average state dynamic route* (TA-SDR) resulting from the earlier arbitrarily specified pulse train stimulation of the nanodevice^16^. A *state dynamic route* (SDR), namely the $\dot{x}$ versus *x* locus, derivable from the state Eq. (1) for a given DC value *V* assigned to the voltage *v*, governs the time evolution of the memory state of a first-order memristor under the specified bias stimulus. In this regard, it is worth to observe that a number of research studies have recently reported laboratory measurements of SDRs acquired from memristive nanodevices, enabling to establish an important communication channel between theoreticians and experimenters. The interested readers are invited to consult works from Messaris^17^, Maldonado^18^, and Marrone^19^ for the details. A TA-SDR can then be interpreted as an extension of the SDR, enabling the investigation of the response of the same device to a particular AC periodic square pulse train. On condition that the choice of the pulse train parameters does not jeopardise the accuracy of the approximation inherent in Eq. (10), the analysis of this new graphic tool enables to determine number, mean values, and stability properties of all the admissible asymptotic oscillations in the memory state of the periodically-forced device. Moreover the predictive capability of the TA-SDR technique may be verified by means of another more rigorous system-theoretic methodology, described shortly in section “The state change per cycle map tool”.

### The state change per cycle map tool

**Figure 3:**
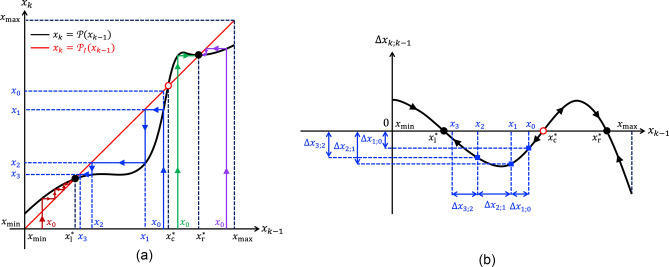
(**a**) Exemplary illustration of a one-dimensional discrete-time system $x_k=\mathscr {P}(x_{k-1})$, referred to as Poincaré map, which admits three intersections with the identity map $x_{k}=\mathscr {P}_I(x_{k-1})=x_{k-1}$, representing its fixed points, specifically $x^*_1$, $x^*_2$, and $x^*_3$, of which the outer ones (inner one) are stable (is unstable). A few coloured zig-zag trajectories, known as *cob-web plots*^12,20^ in Nonlinear Dynamics Theory, are also displayed to show the discrete-time evolution of the map from distinct initial conditions toward one of the two LAS fixed points. In our study a map of this kind can be extracted from the Strachan DAE set, when the input voltage *v* is enforced to follow a given periodic voltage stimulus $v_S$, e.g. in the form of a rectangular pulse train, by recording samples of the memristor state *x* at regular *T*-long time intervals from the beginning of each of a large ensemble of simulations, differing in the initial conditions, and then plotting for each of the resulting time series the *k*th sample $x_k=x(k \cdot T)$ versus the $(k-1)$th one $x_{k-1}=x((k-1) \cdot T)$, with $k \in \mathbb {N}_{>0}$. For $k=1$ the SCPCM reduces to $\Delta _{1;0}=x_1-x_{0} = \mathscr {P}(x_{0})-x_{0}$, providing the change in the memory state over the first input cycle. (**b**) $\Delta x_{k;k-1} = x_k-x_{k-1}$ versus $x_{k-1}$ locus, illustrating the SCPCM of the ReRAM cell subject to the periodic stimulus, which induces a state motion resulting in the Poincaré map shown in plot (**a**).

The State Change Per Cycle Map (SCPCM) analysis tool^20^ is inspired from the Poincaré map technique^12^, a powerful method from Nonlinear Dynamics Theory, which facilitates the study of a *n*th-order non-autonomous periodically-forced continuous-time system, equivalent to a $(n+1)$th-order autonomous continuous-time system, where the time assumes the role of a state variable, through the analysis of a simpler *n*-dimensional discrete-time one. For the Strachan model, featuring order $n=1$, and continuously driven by an input signal *v*, forced to follow a generic periodic voltage stimulus $v_S$, the Poincaré map assumes the one-dimensional form $x_k=\mathscr {P}(x_{k-1})$, where $k \in \mathbb {N}_{>0}$, and $x_k$ stands for the sample $x(k \cdot T)$ of the solution of the ODE (1), with *g*(*x*, *v*) expressed by the formula (3), at the end of the *k*th input cycle. For $k=1$ the map reduces to $x_1=\mathscr {P}(x_0)$, where $x_0$ denotes the ODE initial condition *x*(0), and $x_1$ is the state sample *x*(*T*) at the end of the first input cycle. The Poincaré map accurately provides the sequence of values $x_0, x_1, \ldots$, also referred to as *return points*, extracted from the time series of the ODE solution at regular *T*-long time intervals from the initial instant $t=0$ of the simulation. The one-dimensional discrete-time system is said to admit a *fixed point* $x^*$ if it maps such point into itself, which is mathematically formulated via the equality $x^*=\mathscr {P}(x^*)$ . A fixed point of the map corresponds to a steady-state oscillatory solution for the original non-autonomous continuous-time system. However, the periodic attractor of the non-autonomous ODE (1) is asymptotically stable if and only if the fixed point of the map is also asymptotically stable, which implies the inequality $|\mathscr {P}'(x_{k})|_{x_{k}=x^*}<1$ to hold true. Figure 3a sketches qualitatively how the graph of the map may look like for an exemplary case study, where, similarly as assumed in Fig. 2, a periodic stimulus endows the memory state of the ReRAM cell with two *locally asymptotically stable (LAS)* steady-state oscillatory solutions. A black filled (red hollow) circle is employed to indicate the location of a stable (an unstable) fixed point of the map. For each *k* value in $\mathbb {N}_{>0}$ the SCPCM expresses the net change $\Delta x_{k;k-1} \triangleq x_k-x_{k-1} = \mathscr {P}(x_{k-1})-x_{k-1}$, which the ODE solution *x* undergoes, over the time interval $[(k-1) \cdot T, k \cdot T]$, i.e. within the *k*th input cycle, as a function of its value $x_{k-1}$ at time $t=(k-1) \cdot T$, i.e. either at the end of the $(k-1)$th input cycle, if $k>1$, or at the beginning of the simulation, if $k=1$. The SCPCM admits a graphical visualisation on the $\Delta x_{k;k-1}$ versus $x_{k-1}$ plane, as sketched qualitatively in Fig. 3b, corresponding to the $\mathscr {P}(x_{x-1})$ versus $x_{k-1}$ locus in plot (a) of the same figure. For any initial condition $x_0$ from a set of values uniformly distributed across the state existence domain $\mathscr {D}$, the net change $\Delta x(k;k-1)$ in the state *x* over the time interval $[(k-1) \cdot T, k \cdot T]$ may be marked on this plane at the abscissa corresponding to the state value $x_{k-1}$ at $t=(k-1) \cdot T$ for each $k\in \mathbb {N}_{>0}$. A suitable interpolation method can then be employed to derive the curve, which best fits the sequences of return points collected for all the selected initial conditions. Arrows, pointing to the east (west), are then superimposed along the graph of a SCPCM in the upper (lower) half of the $\Delta x_{k;k-1}$ versus $x_{k-1}$ plane to indicate a progressive step-wise increase (decrease) in the discrete-time evolution of the Poincaré return point when $\Delta x_{k;k-1}$ is positive (negative). For each *k* value in $\mathbb {N}_{>0}$, the $k$th return point $x_k$ of the Poincaré map for a given initial condition $x_0$ may be obtained by adding the abscissa $x_{k-1}$, representing either the $(k-1)$th return point, if $k>1$ or the initial condition, if $k=1$, to the ordinate $\Delta x_{k; k-1}$ of the point of intersection between the graph of the SCPCM and the vertical line passing through the point $(x_{k-1}, 0)$. A fixed point $x^*$ of the Poincaré map corresponds to the state value, at which the SCPCM crosses the $x_{k-1}$ axis, as $\Delta x_{k;k-1}=0$ therein. The stability of a fixed point of the map may be inferred by monitoring the direction of the arrows in its neighbourhood. Arrows, pointing toward (away from) a fixed point on both its left and right sides, provide clear evidence for its asymptotic stability (instability). Alternatively, the same information can be retrieved by inspecting the slope of the graph of the SCPCM at the fixed point. In fact, the fixed point is asymptotically stable (unstable) if and only if the slope of the $\Delta x_{k;k-1}$ versus $x_{k-1}$ locus is negative (positive) at its location.

#### Remark 1

The vector field of the original non-autonomous ODE (1) maps a given state value into some other one over a *T*-long time span, irrespective of the number of input cycles, elapsed since the beginning of the simulation. Therefore, a more efficient strategy to compute a SCPCM, in comparison to the method described earlier, envisages to test the response of the ReRAM cell to a predefined periodic stimulus across a *T*-long time span only, for each initial condition $x_0$ from a set of values uniformly distributed across the state existence domain $\mathscr {D}$. Specifically, in each iteration step, the state value $x_1 \triangleq x(T)$ at the end of a *T*-long simulation, and the state change $\Delta x_{1;0}$, relative to the initial condition, would be recorded, allowing to identify a particular point on the plane, spanned by $x_0$ and $\Delta x_{1;0}$ on the horizontal and vertical axes, respectively. Interpolating the data through some best-fit curve, and renaming the label on the horizontal (vertical) axis as $x_{k-1}$ ($\Delta x_{k;k-1}$) finally results in the graph of the SCPCM of interest.

All in all, the SCPCM technique enables to explore the response of a first-order nonlinear dynamic system to any periodic stimulus. It thus extends the applicability scope of the TA-SDR tool, which is employable solely in those case studies, where a periodic train of rectangular pulses stimulates a system of this kind. Furthermore, following the steps, described in Remark 1, it should be possible to acquire a SCPCM experimentally, provided access to the device state were possible. On the other hand, the measurement of a TA-SDR seems to pose harder challenges. Moreover, as elucidated in this section, *no approximation is involved in the derivation of a SCPCM, which, as a result, may be used to verify the predictions of the TA-SDR investigation technique*. Despite its weak points, however, the latter method allows the derivation of an analytical approach to engineer multi-stability in the oscillatory response of the nano-device to a generalised periodic pulse train voltage stimulus, as described in section “An analytical methodology to operate the pulse-driven ReRAM cell as a multimodal device with initial condition-dependent oscillatory behaviour”. Moreover, upon availability of a reliable model for the ReRAM cell, and for any given rectangular pulse train stimulus, the computation of the SCPCM takes a much longer time than the determination of the respective TA-SDR. In fact, while the latter task simply requires to plot the right hand side of the TA-SE (10), adapted to the excitation signal of interest, against the time average state, the first one requires the numerical integration of the state equation over one input cycle for an adequate number of initial conditions, as explained in Remark 1.

## Insights into the model

Before introducing the analytical framework, allowing to induce mono- or multi-stability in the oscillatory response of the ReRAM cell to a generalised pulse train voltage stimulus, this section discusses numerical investigations, which shed light into the properties of the Strachan model equations as well as into its response to a square wave excitation signal from the class illustrated in Fig. 1b.

### Dynamic route map

**Figure 4:**
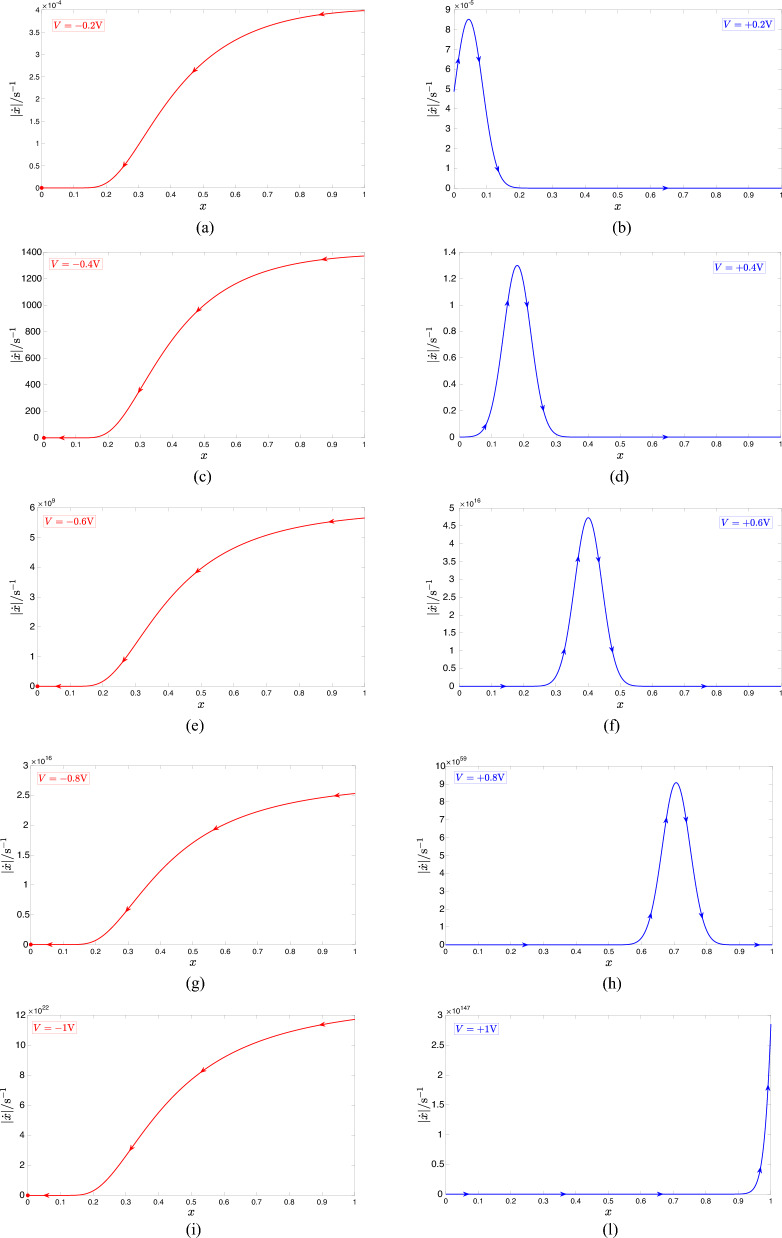
(**a**), (**c**), (**e**), (**g**), (**i**) ((**b**), (**d**), (**f**), (**h**), (**l**)) $g_{\text {RESET}}(x,V)$ ($g_{\text {SET}}(x,V)$) versus *x* locus, denoting the RESET (SET) SDR of the ReRAM cell^15^ when *V* is chosen as the first, second, third, fourth, and fifth value from the set $\{-(+)0.2,-(+)0.4,-(+)0.6,-(+)0.8,-(+)1\}$V. Over a RESET (SET) resistance switching transition the device state undergoes a progressive decrease (increase), as the arrows, superimposed on top of the respective SDR, clearly indicate through their westward (eastward) direction. With reference to each graph along the first column, the red filled circle shows the location of the stable equilibrium $x_{eq}=x_{\text {L}}$, which the ODE (1) admits for any negative bias value *V* assigned to the input voltage *v*. On the other hand, the state equation admits no equilibrium under any positive DC stimulus.

The Dynamic Route Map (DRM) of the first-order ReRAM cell under focus is a family of SDRs, each of which corresponds to the plot of the state evolution function (3) against the state for a particular DC value *V* assigned to the voltage *v*. When *V* is negative (positive), the resulting *g*(*x*, *V*) versus *x* locus is referred to as a *RESET SDR* (*SET SDR*). A number of RESET (SET) SDRs, obtained by sweeping |*V*| in $0.2\, \text{V}$-long steps from $0.2\, \text{V}$ to $1 \, \text{V}$, are shown in plots (a), (c), (e), (g), and (i) ((b), (d), (f), (h), and (l)) of Fig. 4. As may be inferred by inspecting the graphs on the left (right) column of this figure, the choice of the negative (positive) DC value *V* has no significant (a strong) impact on the shape of the resulting RESET (SET) $\dot{x}$ versus *x* locus. We may thus conclude that, toward the development of a strategy to massage the SET $\dot{\bar{x}}|_{\text {SET}}$ and RESET $\dot{\bar{x}}|_{\text {RESET}}$ components of the TA-SE (10) in such a way to enable a desired number of intersections between their graphs, the fine control of the position of the gaussian bell-shaped *g*(*x*, *V*) versus *x* locus across the horizontal axis through smooth changes in the positive DC voltage *V* is worth of exploitation.

### Numerical investigation of the ReRAM response to the basic pulse train stimulus

**Figure 5:**
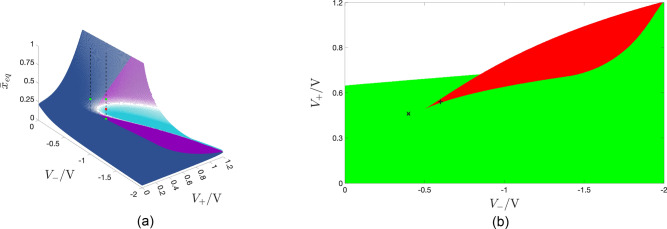
Three-dimensional illustration, showing each admissible stable or unstable equilibrium $\bar{x}_{eq}=\bar{x}_{eq}(V_+,V_-)$, which the TA-SE (10), associated to a train voltage stimulus, featuring two pulses of opposite polarity per cycle, may possibly admit, when the SET $\tau _+$ and RESET $\tau _-$ pulse widths are identical, as a function of the SET $V_+$ and RESET $V_-$ pulse heights, swept across the ranges $[-2,0]$V and $[0,1.2] \, \text{V}$, respectively. The dark blue surface includes all the GAS equilibria of the TA-SE in the monostable oscillatory operating mode of the ReRAM cell. The cyan (magenta) surface contains all the unstable (all the LAS) equilibria of the TA-SE in the bistable oscillatory operating mode of the ReRAM cell. (**b**) Projection of the surface from (**a**) onto the $V_+$ versus $V_-$ plane. Choosing the pulses’ heights of the pulse train voltage stimulus, featuring a $50\%$ duty cycle, according to the coordinates of any point in the green (red) region, the TA-SE features a single GAS equilibrium (two LAS equilibria) for $r=1$. The black cross marker (black plus sign) identifies the input parameter pair $(V_-,V_+)$, inducing the particular monostable (bistable) oscillatory response, illustrated in Fig. 6 (Fig. 7), in the nanodevice.

As uncovered by Pershin and Slipko through an in-depth bifurcation study^8^ of the Strachan model, the ReRAM cell is expected to display one or two LAS oscillatory behaviours in response to the basic pulse train voltage stimulus of Fig. 1b, under the assumption of a unitary SET-to-RESET pulse width ratio $r \triangleq \frac{\tau _+}{\tau _-}$, depending upon the selection of the RESET $V_-$ and SET $V_+$ pulse heights. This is clearly illustrated in Fig. 5a, visualising through a three-dimensional surface each admissible equilibrium $\bar{x}_{eq}=\bar{x}_{eq}(V_+,V_-)$, which the TA-SE (10) admits for $r=1$, endowing the pulse train with a $50\%$ duty cycle, when $V_-$ and $V_+$ are in turn chosen as the abscissa and ordinate of any point of the coloured map in plot (b) of the same figure. The dark blue domain from plot (a) contains the only globally asymptotically stable (GAS) equilibrium $\bar{x}_{\text {eq}}$, which the TA-SE features, upon selecting $(V_-,V_+)$ anywhere within the green region from plot (b). On the other hand, the bottom and top violet domains (the cyan domain) from plot (a) include (includes) the leftmost and rightmost LAS equilibria $\bar{x}_{\text {eq},1}$ and $\bar{x}_{\text {eq},3}$ (the unstable equilibrium $\bar{x}_{\text {eq},2}$) of the TA-SE corresponding to any choice for the input parameter pair within the red domain from plot (b). For the sake of completeness, the white area in the coloured map of plot (b) contains input parameter pairs, whereby there exists no state value, at which $\dot{\bar{x}}|_{\text {SET}}=-\dot{\bar{x}}|_{\text {RESET}}$. In a scenario of this kind, if $\dot{\bar{x}}$ is found to be strictly negative (strictly positive), the memory state of the periodically-forced ReRAM cell shall progressively decrease (increase) toward the lower (upper) bound $x_L$ ($x_U$) in its existence domain $\mathscr {D}$. As the operation of the device around its fully-RESET or fully-SET state is not recommendable, the selection of input parameter pairs, belonging to the white region in the map of plot (b), shall be avoided in the analysis of exemplary excitation case studies to follow.

#### Monostability

**Figure 6:**
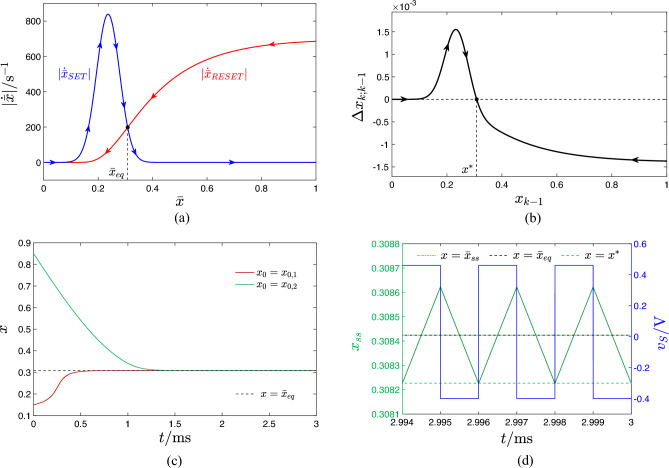
SET $|\dot{\bar{x}}_{\text {SET}}|$ (blue trace) and RESET $|\dot{\bar{x}}_{\text {RESET}}|$ (red trace) components of the TA-SDR of the ReRAM cell under the application of a two-pulse-per-cycle pulse train voltage stimulus $v_S$, when its SET $V_+$ and RESET $V_-$ pulse heights are in turn set to $+0.46 \, \text{V}$ and $-0.4 \, \text{V}$, and for $r=1$, irrespective of the choice of its SET $\tau _+$ and RESET $\tau _-$ pulse widths. Note that scaling the widths of the 2 pulses in the train per cycle by the same factor does not affect the TA-SDR prediction. The only GAS equilibrium $\bar{x}_{eq}$ of the TA-SE lies at 0.308, which is the abscissa of the black-filled circle. A marker, indicating the zero of the RESET component at $\bar{x}=0$, is omitted from the graph, so as to avoid clutter. (**b**) SCPCM of the ReRAM cell subject to a particular pulse train voltage stimulus $v_S$, belonging to the class considered in (**a**), and characterised by parameters $(V_+,\tau _+,V_-,\tau _-)=(+0.46 \, \text{V},1{\upmu \text{s}},-0.4 \, \text{V},1{\upmu \text{s}})$ (refer to the blue signal of period $T=\tau _++\tau _-=2 \, {\upmu \text{s}}$ in plot (**d**)). The Poincaré map, from which it is extracted, features a GAS fixed point $x^*$ (see the black-filled circle). Differently from what is the case for the TA-SDR, scaling the widths of the 2 pulses in the train per cycle by the same factor may affect the SCPCM. (**c**) Brown (Green) trace: progressive approach of the solution *x* to the Strachan DAE set, when *v* is forced to follow the particular excitation voltage signal $v_S$, employed for the derivation of the SCPCM, from the initial condition $x_0=x_{0,1}=0.15$ ($x_0=x_{0,2}=0.85$) toward a unique steady-state oscillation. (**d**) Green trace: steady-state time series $x_{\text {ss}}$ of the memristor state *x*, as extracted from the solution featuring the same colour in plot (**c**). Horizontal lines mark the locations of the map fixed point $x^*$, of the TA-SE equilibrium $\bar{x}_{eq}$, and of the time average $\bar{x}_{\text {ss}}$ of the steady-state time series. As the RESET pulse follows the SET pulse over each cycle of the input train, $x_{\text {ss}}$ attains its minimum value at the end of any period. Therefore $x^*$ directly reveals the minimum of $x_{\text {ss}}$ across one input cycle.

Taking $V_-$ and $V_+$ in turn as the abscissa and ordinate of the point $(-0.4 \, \text{V},+0.46 \, \text{V})$, indicated as a black cross marker, and belonging to the green region in the map of Fig. 5b, as may be inferred from Fig. 6a, showing the $|\dot{\bar{x}}|_{\text {SET}}|$ and $|\dot{\bar{x}}|_{\text {RESET}}|$ versus $\bar{x}$ loci for the earlier specified $(V_-,V_+)$ pair, the TA-SDR analysis predicts a monostable oscillatory behaviour for the periodically-forced ReRAM cell. Its memory state *x* is expected to experience a steady-state oscillation around the TA-SE equilibrium $\bar{x}_{eq}=0.308$. With reference to plot (a) of Fig. 5, the left vertical black dashed line crosses the blue domain of the three-dimensional surface in a single point, specifically $(V_-,V_+,\bar{x}_{\text {eq}})=(-0.4 \, \text{V},+0.46 \, \text{V},0.308)$, as indicated through a green filled circle. Choosing sufficiently small values for the RESET $\tau _-$ and SET $\tau _+$ pulse widths is instrumental to prevent the error in the approximation inherent in Eq. (10) to jeopardise the accuracy of its predictions. Fixing both $\tau _-$ and $\tau _+$ to $1{\upmu \text{s}}$, the SCPCM of the ReRAM cell, shown in Fig. 6(b), confirms the conclusions drawn via TA-SDR analysis from plot (a) of the same figure. Figure 6c shows the progressive approach of the solution *x* to the state Eq. (1) toward the only possible asymptotic periodic waveform, revolving approximately around $\bar{x}_{\text {eq}}=0.308$, from either of two initial conditions, lying one well below the minimum and the other well above the maximum of the steady-state oscillation. Plot (d) of the same figure visualises both the periodic pulse train voltage stimulus $v_S$ (in blue) and the steady-state oscillation $x_{\text {ss}}$ in the memristor state (in green) together with its mean value $\bar{x}_{\text {ss}}$, its prediction, namely the TA-SE equilibrium $\bar{x}_{\text {eq}}$, as well as the map fixed point $x^*$, corresponding to the minimum value it assumes over each cycle.

#### Bistability

**Figure 7:**
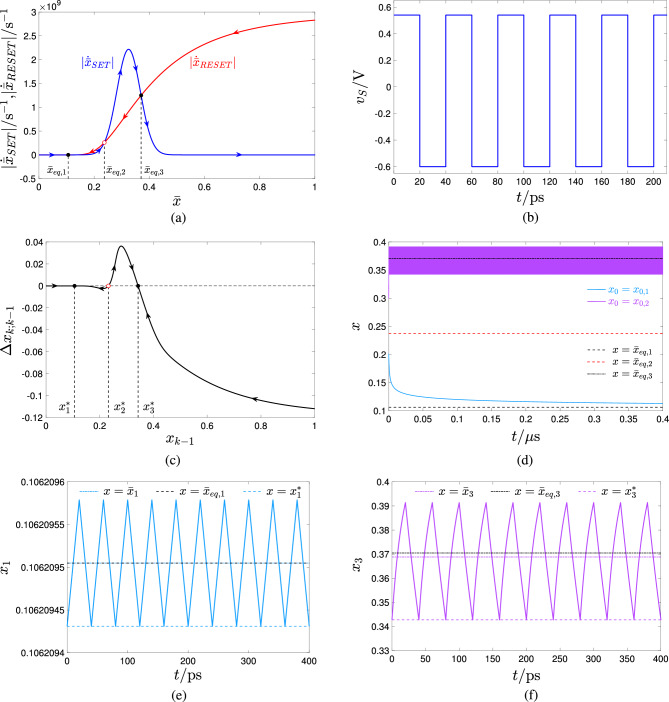
Decomposition of the TA-SDR into its SET $|\dot{\bar{x}}_{\text {SET}}|$ (blue trace) and RESET $|\dot{\bar{x}}_{\text {RESET}}|$ (red trace) components—plot (a)—for the ReRAM cell subject to a two pulse-per-cycle pulse train voltage stimulus $v_S$, composed of one SET (RESET) pulse of positive (negative) amplitude $V_+=+0.54 \, \text{V}$ $(V_-=-0.6 \, \text{V})$ over the first (second) $\tau _+(\tau _-)$-long half of each period of duration $T=\tau _++\tau _-$, irrespective of the common value assigned to $\tau _+$ and $\tau _-$. The TA-SE admits a triplet of equilibria, namely $\bar{x}_{eq,1}=0.106$, $\bar{x}_{eq,2}=0.237$, and $\bar{x}_{eq,3}=0.370$. Each of the outer ones (The inner one), indicated via a black-filled (red hollow) circle, is LAS (unstable). (**b**) Time waveform of a particular pulse train voltage stimulus, belonging to the class assumed in (**a**), and identified via the parameter quartet $(V_+,\tau _+,V_-,\tau _-)=(+0.54 \, \text{V},20 \, \text{ps},-0.6 \, \text{V},20 \, \text{ps})$. (**c**) SCPCM of the ReRAM cell in the case, where the excitation voltage signal $v_S$ from (**b**) is let fall continuously between its terminals. A black-filled (red hollow) circle denotes a locally-stable (an unstable) fixed point for the associated Poincaré map. (**d**) Cyan (Violet) trace: time course of the memory state *x* of the ReRAM cell, with voltage *v* forced to follow $v_S$ from (**b**) at all times, from the initial condition $x=x_{0,1}=0.2$ ($x=x_{0,2}=0.3$). Unlike the latter solution, the first one takes a very long time to attain the steady state. (**e**, **f**) Locally-stable oscillatory solution $x_1$ ($x_3$) for the state *x* of the ReRAM cell, as recorded in a numerical simulation of the Strachan DAE set under $v=v_S$ from (**b**) for $x_0=x_1^*$ ($x_0=x_3^*$). In each of the two cases the choice of the initial condition ensures that no transients appear in the device response. The time average $\bar{x}_1$ ($\bar{x}_3$) of the solution $x_1$ ($x_3$), as well as the corresponding LAS TA-SE equilibrium $\bar{x}_{eq,1}$ ($\bar{x}_{eq,3}$) and LAS map fixed point $x^*_1$ ($x^*_3$) are also marked in plot (**e**, **f**).

Setting the RESET $V_-$ and SET $V_+$ pulse heights to $-0.6\text{V}$ and $+0.54\text{V}$, respectively, which identifies a point, indicated through a black plus sign, and lying within the red region in the map of Fig. 5b, the TA-SDR analysis predicts the coexistence of two LAS steady-state oscillatory solutions for the memory state *x* of the periodically-excited ReRAM cell, as may be inferred from Fig. 7a, revealing the existence of a triplet of crossings between the loci of the moduli of the SET and RESET TA-SE components. The abscissa of each of the two outer intersections $\bar{x}_{eq,1}=0.106$ and $\bar{x}_{eq,3}=0.370$ (of the inner intersection $\bar{x}_{eq,2}=0.237$) denotes a LAS (an unstable) equilibrium for Eq. (10). The right vertical black dashed line in Fig. 5a intersects the bottom and top violet domains in the green-filled points $(V_-,V_+,\bar{x}_{eq})=(-0.6 \, \text{V},+0.54 \, \text{V},0.106)$ and $(V_-,V_+,\bar{x}_{eq})=(-0.6 \, \text{V},+0.54 \, \text{V},0.370)$, respectively, and the cyan domain in the red-filled point $(V_-,V_+,\bar{x}_{eq})=(-0.6 \, \text{V},+0.54 \, \text{V},0.237)$. Setting the RESET $\tau _-$ and SET $\tau _+$ pulse widths to a relatively small value, specifically $40 \, \text{ps}$, as shown in plot (b) of Fig. 7, visualising the time waveform of the resulting train voltage stimulus $v_S$, allows to limit the change in the memory state over each cycle, which endows the TA-SDR graphic tool with predictive capability. In fact, as may be inferred from plot (c) of the same figure, the SCPCM of the ReRAM cell, subject to the stimulus from plot (b), validates the conclusions drawn through the analysis of the TA-SE (10). The cyan (violet) trace in Fig. 7d depicts the transient behaviour of a solution to the ODE (1), as it approaches the LAS oscillatory waveform revolving approximately around the leftmost (rightmost) TA-SE equilibrium $\bar{x}_{eq,1}$ ($\bar{x}_{eq,3}$). Due to the slow/fast dynamical effects, emerging in the nanodevice, it takes a rather long (rather short) time for the first (second) solution to attain the steady state. However, an ad hoc choice of the initial condition may allow to retrieve the asymptotic behaviour of the state of the periodically-driven ReRAM cell without the need to wait for transients to vanish. In fact, plot (e) ((f)) of the same figure illustrates the transient-free solution $x_1$ ($x_3$) to the ODE (1), initiated from the leftmost (righmost) map fixed point $x^*_1$ ($x^*_3$), corresponding to the minimum value the state assumes over each cycle, together with its mean value $\bar{x}_1$ ($\bar{x}_3$), and the respective approximation $\bar{x}_{eq,1}$ ($\bar{x}_{eq,3}$).

#### On the crossings between one scaled SET SDR and one scaled RESET SDR

**Figure 8:**
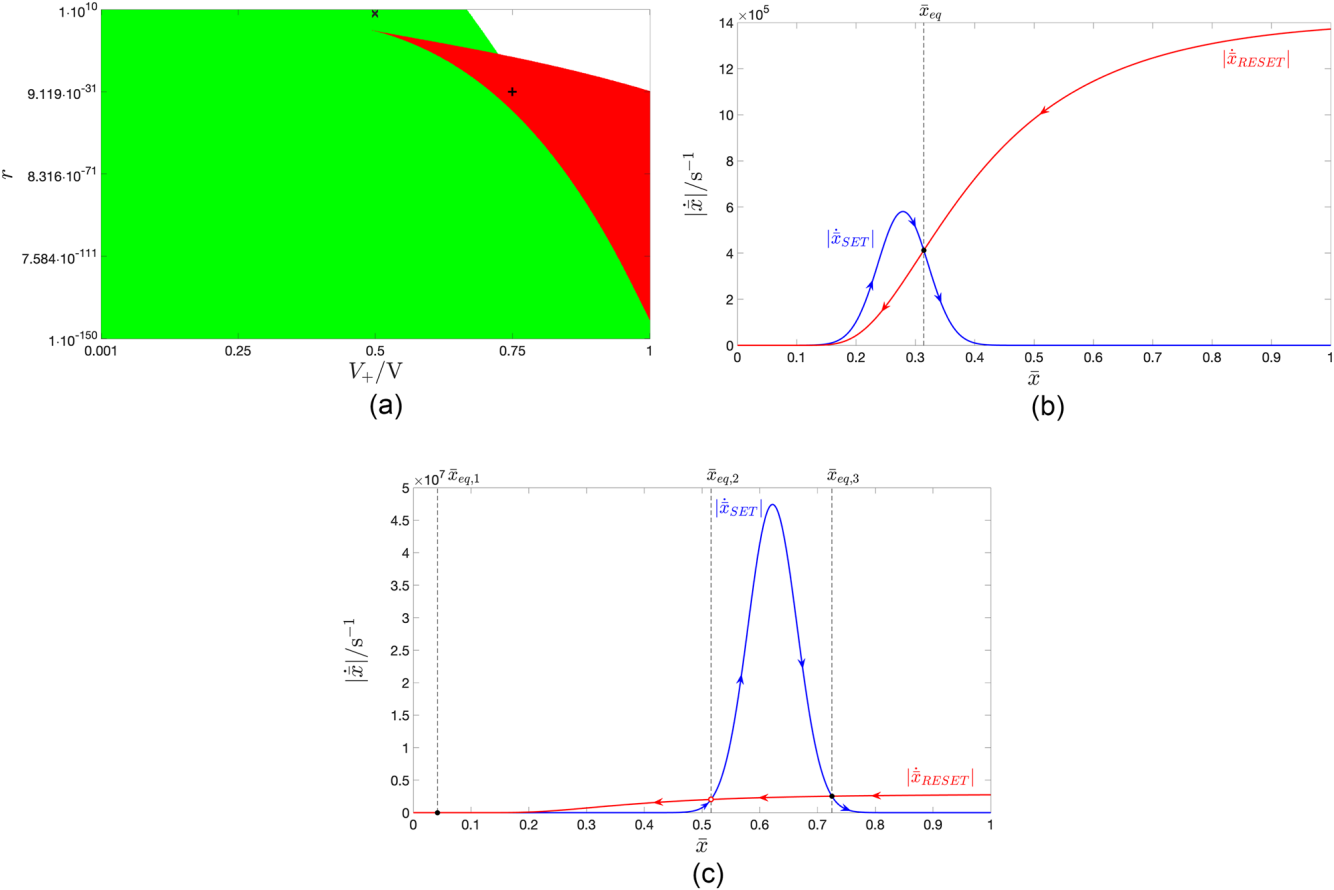
(**a**) Coloured map, depicting how the number of admissible stable or unstable equilibria for the TA-SE of the ReRAM cell, subject to a two-pulse-per-cycle pulse train voltage stimulus from the class illustrated in Fig. 1b is influenced by the SET pulse amplitude $V_+$ as well as by the ratio *r* between the SET and RESET pulse widths, given a RESET pulse amplitude $V_-$ of $-0.5 \, \text{V}$. The green and red regions respectively enclose input parameter pairs, which endow the TA-SE with one and only one GAS equilibrium (three equilibria, of which the outer ones are LAS). (**b**, **c**) Graphical illustration, showing the decomposition of the TA-SDR into its SET and RESET components for a scenario, where the input pair $(V_+,r)$, lying at $(+0.50 \, \text{V},1 \times 10^{8})$ $((+0.75 \, \text{V},1 \times 10^{-30}))$ (see the black cross marker (black plus sign) within the green (red) region of the map in (**a**)), determines the existence of one and only one GAS equilibrium $\bar{x}_{eq}=0.314$ (three equilibria $\bar{x}_{eq,1}=0.042$, $\bar{x}_{eq,2}=0.516$, and $\bar{x}_{eq,3}=0.725$, of which the outer ones are LAS) for the respective TA-SE.

In general, a single gaussian bell-shaped SET SDR may cross a single RESET SDR nowhere, which is of no practical interest, as elucidated in section “Numerical investigation of the ReRAM response to the basic pulse train stimulus”, in one point, endowing the resulting TA-SE with a GAS equilibrium $\bar{x}_{\text {eq}}$, or in three locations, specifically $\bar{x}_{\text {eq},1}$, $\bar{x}_{\text {eq},2}$, and $\bar{x}_{\text {eq},3}$, the outer of which denote LAS equilibria for the corresponding TA-SE. For a fixed choice of the negative pulse height $V_-$, this depends upon the amplitude $V_{+}$ of the positive pulse as well as upon the SET-to-RESET pulse width ratio *r*, as may be inferred from the coloured map, shown in Fig. 8a, which was derived by means of a numerical procedure for $V_-=-0.5 \, \text{V}$, and depicts through a white, a green, and a red hue the regions of the *r* versus $V_+$ plane, where, according to the TA-SDR analysis, the ReRAM cell is expected to admit no, a monostable, and a bistable oscillatory behaviour at steady state, respectively. Fig. 8b, c) illustrates the loci of the SET and RESET TA-SE components for a choice of the input parameter pair $(V_+,r_{+})$, specifically $(+0.50 \, \text{V},1 \times 10^{8})$ $((+0.75 \, \text{V},1 \times 10^{-30}))$ (see the black cross marker (black plus sign) within the green (red) domain in plot (a) of the same figure), which is expected to trigger a monostable (bistable) oscillatory response in the ReRAM cell, when $V_-$ is fixed to $-0.5 \, \text{V}$.

## An analytical methodology to operate the pulse-driven ReRAM cell as a multimodal device with initial condition-dependent oscillatory behaviour

Despite an in-depth numerical investigation may allow to explore the response of the ReRAM cell to a rectangular pulse train stimulus, the availability of an analytical strategy to craft the excitation signal so as to endow the memory state of the ReRAM cell with a prescribed number of steady-state oscillatory solutions, revolving around predefined levels, would be of greater interest for circuit designers. In order to address this point, this section first presents a thorough analytical investigation of the Strachan model, and then employs its findings to propose a systematic approach to engineer multistability in the oscillatory response of the ReRAM cell to a generalised pulse train voltage stimulus from the class defined in the section to follow.

### Adaptation of the TA-SE to a generalised pulse train stimulus

In fact, here the periodic voltage source $v_S$ in the test circuit of Fig. 1a is assumed to emit a generalised pulse train from the class illustrated in Fig. 1c. In each cycle the generalised train is composed of a tunable number $P \in \mathbb {N}_{>0}$ of positive SET pulses followed by a single RESET pulse. Let the *i*th SET pulse feature a height $V_{+,i}$ and a width $\tau _{+,i}$, with $i\in \{1,2,\ldots ,P\}$. The height and width of the RESET pulse are indicated as $V_-$ and $\tau _-$, respectively. The input period is thus computable as $T=\tau _{+,1}+\tau _{+,2}+\ldots +\tau _{+,P}+\tau _{-}$. Under this hypothesis, Eq. (8) may be expanded as

13 $$\begin{aligned} \dot{\bar{x}}= & {} \frac{1}{T} \cdot \int _{t}^{t + \tau _{+,1}} g_{\text {SET}}(x(t'), V_{+,1}) dt'+\frac{1}{T} \cdot \sum _{j=2}^{P} \int _{t+\tau _{+,j-1}}^{t + \tau _{+,j}} g_{\text {SET}}(x(t'), V_{+,j}) dt'+\frac{1}{T} \cdot \int _{t+T-\tau _-}^{t + T} g_{\text {RESET}}(x(t'), V_-) dt'. \end{aligned}$$

Let us further assume each pulse in the train of Fig. 1c to induce a negligible change in the device memory state. This allows to approximate the state *x*, appearing in each integrand function from Eq. (13) with its time average state $\bar{x}$, allowing to derive the TA-SE of the ReRAM cell, subject to the generalised train voltage stimulus. In its approximate formula, still provided by Eq. (10), the RESET component keeps the expression reported in (12), while the SET component reads as

14 $$\begin{aligned} \dot{\bar{x}}_{\text {SET}}= & {} \sum _{i=1}^{P} \tilde{\tau }_{+,i} \cdot g_{\text {SET}}(\bar{x}, V_{+,i}), \end{aligned}$$

with $$\tilde{\tau }_{+,i} \triangleq \tau _{+,i}/T$$.

### Extraction of key geometrical features from a gaussian bell-shaped SET state evolution function

The proposed strategy to endow multistability in the oscillatory response of the ReRAM cell to a generalised pulse train envisages an ad hoc choice for the $P+1$ stimulus parameters $(V_{+,1},\tau _{+,1},V_{+,2},\tau _{+,2},\ldots ,V_{+,P},\tau _{+,P},V_{-},\tau _{-})$, for $P \in \mathbb {N}_{>0}$, so as to shape the locus of the SET TA-SE component $\dot{\bar{x}}_{\text {SET}}$ in such a way to let it intersect the locus of the RESET TA-SE component $\dot{\bar{x}}_{\text {SET}}$, while keeping above (below) it to the left (right) of the crossing, in as many locations as specified in the design requirements. In fact, *it is fundamental to devise an ad hoc linear combination between scaled gaussian bell-shaped SET state evolution functions for massaging the SET TA-SE component according to the design specifications.* The derivation of a few key geometrical properties of a generic gaussian bell-shaped SET SDR, i.e. the $g_{\text {SET}}(x,v)$ versus *x* locus, resulting from assigning an arbitrary positive DC value $V_+$ to the voltage *v* (recall the graphs along the right column of Fig. 4), is instrumental to accomplish this goal.

#### State value at the peak of a SET SDR

This section derives an exact closed-form expression for the state value, at which a generic gaussian bell-shaped SET SDR attains its peak level. Employing Eqs. (1), (3), and (4), the rate of change $\dot{x}$ of the memory state *x* under the application of a positive bias voltage $V_+$ across the device may be cast as

15 $$\begin{aligned} \dot{x}=g_{\text {SET}}(x,V_+)=B \cdot \sinh \left( \frac{V_+}{\sigma _{\text {on}}}\right) \cdot \exp \left( \alpha (x,V_+)\right) , \end{aligned}$$

where

16 $$\begin{aligned} \alpha (x,V_+) \triangleq -\frac{x^2}{x_{\text {on}}^2}+\frac{G(x,V_+) \cdot V_+^2}{\sigma _{\text {p}}}, \end{aligned}$$

with $G(x,V_+)$ expressing the dependence of the device conductance upon its state under the prescribed positive DC stimulus, according to Eq. (6). The abscissa of the maximum of the $\dot{x}$ versus *x* locus for $v=V_+$ may be analytically computed by employing Eq. (15), and finding the state value, at which $\partial g_{\text {SET}}(x,V_+)/ \partial x$ vanishes. As the exponential function on the right hand side of Eq. (15) is a monotonically increasing function of its argument, it is sufficient to find the state value, at which $\partial \alpha (x,V_+)/\partial x$ vanishes. After some algebraic calculation, the formula for $x_{\text {max}}(V_+)$ is found to read as

17 $$\begin{aligned} x_{\text {max}}(V_+) =\frac{V_+^2 \cdot x_{\text {on}}^2}{2 \cdot \sigma _{\text {p}}} \cdot \gamma (V_+), \end{aligned}$$

where

18 $$\begin{aligned} \gamma (V_+) \triangleq G_{\text {m}}-a \cdot \exp \left( \,b \cdot \sqrt{V_+} \right) . \end{aligned}$$

**Figure 9:**
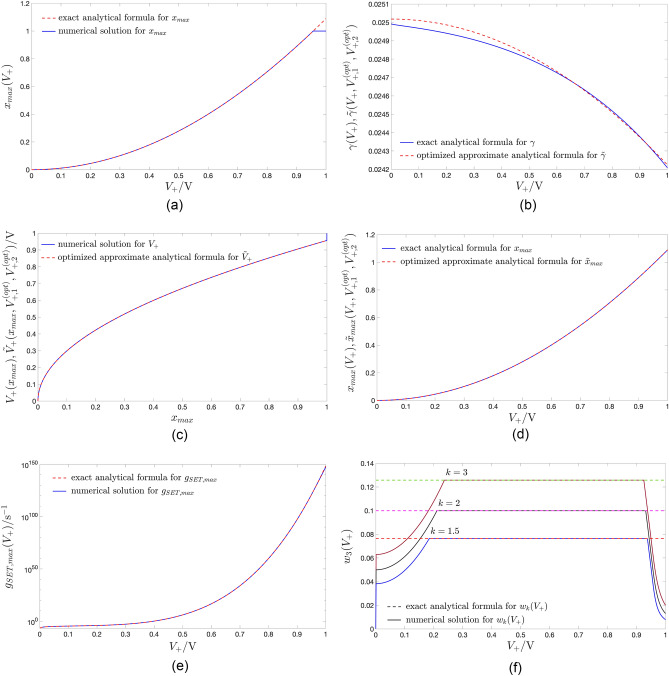
(**a**) Dependence of the abscissa $x_{\text {max}}$ of the peak of the gaussian bell, illustrating a SET SDR, i.e. a $g_{\text {SET}}(x,V)$ versus *x* locus from the ReRAM cell DRM (refer for examples to plots (**b**), (**d**), (**f**), (**h**), and (**l**) of Fig. 4), upon the positive DC voltage $V=V_+\in [0,1] \, \text{V}$. The exact analytical solution, descending from the formula (17), is illustrated in red. The numerical solution, depicted in blue, saturates abruptly to the unitary value at the first positive DC voltage $V_+$, specifically $0.957 \, \text{V}$, where $x_{\text {max}}$ exceeds the upper bound $x_{\text {U}}$ of the state existence domain $\mathscr {D}$, keeping unchanged for any larger $V_+$ value. (**b**) Blue trace: Graph of $\gamma$ as a function of $V_+$, according to the exact analytical formula (18). Red trace: approximation of the $\gamma$ versus $V_+$ locus via the analytical function $\tilde{\gamma }(V_+,V_{+,1},V_{+,2})$ from Eq. (19) for $(V_{+,1},V_{+,2})=(V^{(\text {opt})}_{+,1},V^{(\text {opt})}_{+,2})=(0.662,0.923)\text{V}$. (**c**) Positive value $V_+$ to be assigned to the DC voltage *V* in order for the abscissa of the peak of the resulting SET SDR to lie at a pre-specified location $x_{\text {max}}$. The blue curve shows the $V_+$ versus $x_{\text {max}}$ locus determined numerically from the blue-coloured numerical solution in (**a**) by exchanging the data series reported along horizontal and vertical axes. At $x_{\text {max}}=1$ the blue trace abruptly turns into a vertical segment stretching from $V_+=0.957 \, \text{V}$ to $V_+=1 \, \text{V}$. The red curve is the $\tilde{V}_+$ versus $x_{\text {max}}$ locus, extracted from the analytical formula (22), proposed to approximate the inverse of the function (17), for $V_{+,1}=V^{(\text {opt})}_{+,1}$, and $V_{+,2}=V^{(\text {opt})}_{+,2}$. (**d**) Blue trace: graphical illustration of the exact analytical formula (17) for $x_{\text {max}}$. Red trace: $\tilde{x}_{\text {max}}$ versus $V_+$ locus, obtained from the approximate closed-form expression (20) for $(V_{+,1},V_{+,2})=(V^{(\text {opt})}_{+,1},V^{(\text {opt})}_{+,2})$. (**e**) Peak value $g_{\text {SET, max}}(V_+)$ of a SET SDR as a function of the positive DC voltage $V_+$ across the ReRAM cell. The red trace shows the exact analytical solution, derived from the closed-form expression (27), while the blue trace depicts its numerical counterpart. (**f**) Impact of the positive DC voltage $V_+$ on the width $w_k(V_+)$ of the respective SET SDR, measured as the distance between the state values $x_{+,k}$ and $x_{-,k}$, at which $g_{\text {SET}}(x,V_+)$ appears to be scaled down by a factor *k* as compared to its peak value $g_{\text {SET}}(x_{\text {max}},V_+)$, for each *k* value from the set $\{1.5,2,3\}$. The exact analytical solution, descending from the formula (35), (The numerical solution) is illustrated through a dashed (solid) trace with red (blue), magenta (black), and green (brown) hue for the first, second, and third *k* value from the triplet. When 1.5, 2, and 3 is assigned to *k*, the numerical solution deviates from the corresponding analytical one as soon as *V* descends below $+0.184$, $+0.211$, $+0.237 \, \text{V}$ (increases above $+0.937$, $+0.932$, and $+0.925 \, \text{V}$), since then $x_-$ ($x_+$) descends below (rises above) the lower (upper) bound $x_L$ ($x_U$) in the state existence domain $\mathscr {D}$.

Figure 9a shows the $x_{\text {max}}$ versus $V_+$ locus, extracted from the formula (17) (red trace) together with its numerical approximation (blue trace).

**Figure 10:**
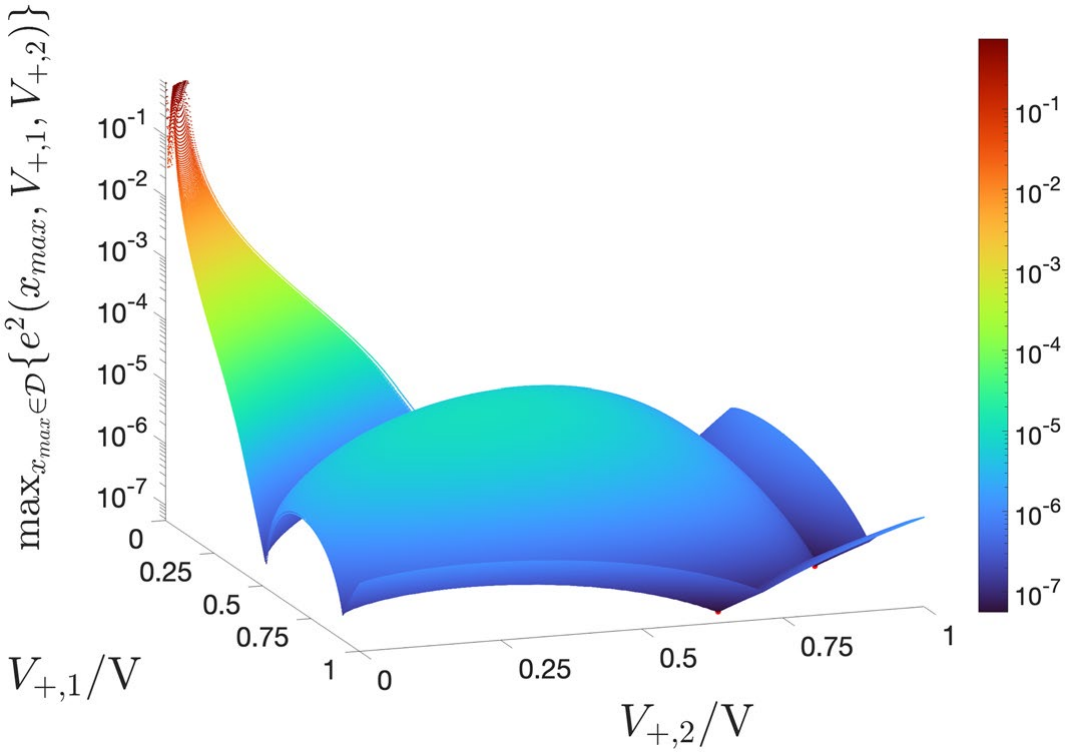
Surface of the maximum squared error $\max _{x_{\text {max}}\in \mathscr {D}}\{e^2(x_{\text {max}},V_{+,1},V_{+,2})\}$ as a function of the voltage parameters $V_{+,1}$ and $V_{+,2}$ under optimisation. At each of the points $(V_{+,1},V_{+,2})=(0.662,0.923) \, \text{V}$ and $(V_{+,1},V_{+,2})=(0.923,0.662) \, \text{V}$, marked as red circles, and symmetrically located relative to the plane $V_{+,2}=V_{+,1}$, the surface assumes the minimum possible value, specifically $5.635 \times 10^{-8}$. Without loss of generality, in the remainder of this paper $V_{+,2}$ is assumed to be larger than $V_{+,1}$. As a result the optimal parameter pair is chosen as $(V^{\text {(opt)}}_{+,1},V^{\text {(opt)}}_{+,2})=(0.662,0.923) \, \text{V}$.

#### Positive DC voltage for programming the abscissa of the peak of a SET SDR

This section derives an approximate analytical formula for the positive bias voltage $V_+$ to be assigned to the voltage *v* for the respective $\dot{x}=g_{\text {SET}}(x,v)$ versus *x* locus to feature the peak level at a preliminarily specified state value $x_{\text {max}}$. The function in Eq. (17) may not be inverted analytically, which explains the reason for the search of a suitable approximate formula. The solid blue trace in Fig. 9b shows the dependence of the function $\gamma (V_+)$ upon $V_+$, as it descends from the exact closed-form expression (18). Let us approximate the expression for $\gamma (V_+)$ with a quadratic polynomial of the form

19 $$\begin{aligned} \tilde{\gamma }(V_+,V_{+,1},V_{+,2})=a_0(V_{+,1},V_{+,2})+a_1(V_{+,1},V_{+,2}) \cdot V_+^2, \end{aligned}$$

where $a_0(V_{+,1},V_{+,2})$ and $a_1(V_{+,1},V_{+,2})$ are functions of two voltage parameters, specifically $V_{+,1}$ and $V_{+,2}$, allowed to range between $0 \, \text{V}$ and $1 \, \text{V}$, and shortly subject to an optimisation procedure. Importantly, $a_0$ and $a_1$ are strictly positive- and negative-valued, respectively, since, as may be evinced by inspecting the blue trace in Fig. 9b, the original function $\gamma (V_+)$ features a positive polarity for $V_+=0\,\text{V}$, and admits a downward concavity, respectively. Replacing $\gamma (V_+)$ with $\tilde{\gamma }(V_+,V_{+,1},V_{+,2})$ into the formula (17) for $x_{\text {max}}$ delivers an approximate analytical formula for the abscissa of the peak of the gaussian bell-shaped SET SDR, reading as

20 $$\begin{aligned} \tilde{x}_{\text {max}}(V_+,V_{+,1},V_{+,2}) =\frac{V_+^2 \cdot x_{\text {on}}^2}{2 \cdot \sigma _{\text {p}}} \cdot \tilde{\gamma }(V_+,V_{+,1},V_{+,2}). \end{aligned}$$

Inserting now the second-order polynomial (19) in place for $\tilde{\gamma }(V_+,V_{+,1},V_{+,2})$ into this equation yields the biquadratic equation

21 $$\begin{aligned} V_+^4 + \frac{a_0(V_{+,1},V_{+,2})}{a_1(V_{+,1},V_{+,2})} \cdot V_+^2 - \frac{2 \cdot \sigma _{\text {p}} \cdot x_{\text {max}}}{x^2_{\text {on}} \cdot a_1(V_{+,1},V_{+,2})} = 0, \end{aligned}$$

which can be solved for $V_+$, resulting in an approximate analytical formula for $V_+(x_{\text {max}})$, featuring the form

22 $$\begin{aligned} \tilde{V}_+(x_{\text {max}},V_{+,1},V_{+,2}) = +\sqrt{-\frac{a_0(V_{+,1},V_{+,2})}{2 \cdot a_1(V_{+,1},V_{+,2})}-\sqrt{\left( \frac{a_0(V_{+,1},V_{+,2})}{2 \cdot a_1(V_{+,1},V_{+,2})}\right) ^2+\frac{2 \cdot \sigma _{\text {p}} \cdot x_{\text {max}}}{x_{\text {on}}^2 \cdot a_1(V_{+,1},V_{+,2})}}}, \end{aligned}$$

in which the positive (negative) sign in front of the first (second) square root sign descends from the polarity of $V_+$ (from the monotonic increase of $V_+$ with $x_{\text {max}}$, as inferable from the graphs along the right column of Fig. 4), and where the functions $a_0(V_{+,1},V_{+,2})$ and $a_1(V_{+,1},V_{+,2})$ are in turn defined as

23 $$\begin{aligned} a_0(V_{+,1},V_{+,2})\triangleq & {} \frac{\tilde{\gamma }(V_{+,1}) \cdot V^2_{+,2} - \tilde{\gamma }(V_{+,2}) \cdot V^2_{+,1}}{V^2_{+,2}-V^2_{+,1}}, \ \text {and} \end{aligned}$$

24 $$\begin{aligned} a_1(V_{+,1},V_{+,2})\triangleq & {} \frac{\tilde{\gamma }(V_{+,2})-\tilde{\gamma }(V_{+,1})}{V^2_{+,2}-V^2_{+,1}}. \end{aligned}$$

Let us define the error *e* between $x_{\text {max}}$ and its approximation $x_{\text {max}}(\tilde{V}_+(x_{\text {max}}),V_{+,1},V_{+,2})$ as

25 $$\begin{aligned} e(x_{\text {max}},V_{+,1},V_{+,2}) \triangleq x_{\text {max}}-x_{\text {max}}(\tilde{V}_+(x_{\text {max}},V_{+,1},V_{+,2}), \end{aligned}$$

in which the second addend on the right hand side calls for the use of the approximate formula $\tilde{V}_+(x_{\text {max}},V_{+,1},V_{+,2})$ for $V_+(x_{\text {max}})$, as given in Eq. (22), into the closed-form expression for $x_{\text {max}}(V_+)$, reported in Eq. (17), with $\gamma (\cdot )$ denoting the function in (18). For each voltage parameter pair $(V_{+,1},V_{+,2})$, we first computed the maximum of the squared error $e^2$, as $x_{\text {max}}$ was swept in $\mathscr {D}$, and then found the minimum of the resulting list of numbers via

26 $$\begin{aligned} \min _{V_{+,1},V_{+,2} \in [0,1]\text{V}}\{\max _{x_{\text {max}} \in \mathscr {D}}\{e^2(x_{\text {max}},V_{+,1},V_{+,2})\}\}. \end{aligned}$$

According to this optimisation procedure, assuming $V_{+,2}>V_{+,1}$, the best voltage parameter pair $(V_{+,1},V_{+,2})$, let us denote it as $(V^{(\text {opt})}_{+,1},V^{(\text {opt})}_{+,2})$, was found to be equal to $(0.662,0.923) \, \text{V}$, which delivered the lowest possible maximum squared error, amounting to $5.635 \times 10^{-8}$. (see the rightmost red-filled circle in Fig. 10). The complementary hypothesis $V_{+,2}<V_{+,1}$ results in an optimal voltage parameter pair $(V^{(\text {opt})}_{+,1},V^{(\text {opt})}_{+,2})=(0.923,0.662) \, \text{V}$, whereby once again the maximum squared error descends to its global minimum (see the leftmost red-filled circle in Fig. 10). Using these optimal values for $V_{+,1}$ and for $V_{+,2}$, the formulas for $\tilde{\gamma }(V_+,V_{+,1},V_{+,2})$ and for $\tilde{V}_+(x_{\text {max}},V_{+,1},V_{+,2})$ are respectively plotted through red traces in Fig. 9b and c. In the latter plot the blue curve illustrates the numerical approximation for the inverse of the function in Eq. (17). Figure 9d depicts the approximate analytical formula (20) for $x_{\text {max}}$ under $V_{+,1}=V^{(\text {opt})}_{+,1},$ and $V_{+,2}=V^{(\text {opt})}_{+,2}$ (red trace) together with the exact analytical closed-form expression for $x_{\text {max}}$ from (17) (blue trace).

#### Peak value of a SET SDR

The maximum value attained by the $\dot{x}$ versus *x* locus for a given positive bias level $V_+$ assigned to the voltage *v* may be easily derived by inserting the analytical closed-form expression (17), with $\gamma (V_+)$ expressed via Eq. (18), in place for $x_{\text {max}}$ on the right hand side of (15), which employs (16) for $\alpha (x,V_+)$. Algebraic manipulations allow to express the maximum $g_{\text {SET, max}}(V_+)$ of the SET state evolution function $g_{\text {SET}}(x,V_+)$ for a given choice of $V_+$ as

27 $$\begin{aligned} g_{\text {SET, max}}(V_+) \triangleq g_{\text {SET}}(x_{\text {max}}(V_+),V_+)=B \cdot \sinh \left( \frac{V_+}{\sigma _{\text {on}}}\right) \cdot \exp \left( \alpha (x_{\text {max}}(V_+),V_+)\right) , \end{aligned}$$

where

28 $$\begin{aligned} \alpha (x_{\text {max}}(V_+),V_+)=\frac{x_{\text {on}}^2 \cdot V_+^4}{4 \cdot \sigma _{\text {p}}^2} \cdot \left( G_{\text {m}}-a \cdot \exp \left( b \cdot \sqrt{V_+} \right) \right) ^2+\frac{a \cdot V_+^2}{\sigma _{\text {p}}} \cdot \exp \left( b \cdot \sqrt{V_+}\right) . \end{aligned}$$

Figure 9e shows the $g_{\text {SET, max}}(V_+)$ versus $V_+$ locus, as extracted from the exact analytical formula (27) (red trace) and by means of a numerical procedure (blue trace).

#### Width of the Gaussian bell-shaped SET state evolution function

For any positive DC value $V_+$ assigned to the voltage *v*, the SET state evolution function $g_{\text {SET}}(x,V_+)$ features a gaussian shape on the $\dot{x}$ versus *x* plane. Let $x_-(V_+)$ and $x_+(V_+)$ denote two state values, lying to the left and to the right of the abscissa of the maximum $x_{\text {max}}(V_+)$ of the gaussian function, respectively. Assume $x_{-,k}(V_+)$ and $x_{+,k}(V_+)$ to hold the same distance from $x_{\text {max}}(V_+)$, and the common value of the SET state evolution function at each of these points to appear scaled down by a factor *k* relative to the maximum level $g_{\text {SET,max}}(V_+)$, which may be expressed in mathematical terms as

29 $$\begin{aligned} g_{\text {SET}}(x_{-,k}(V_+),V_+)=g_{\text {SET}}(x_{+,k}(V_+),V_+)=\frac{1}{k} \cdot g_{\text {SET,max}}(V_+). \end{aligned}$$

For a given choice of $k\in \mathbb {R}$, let us now define the *k*th-scale width $w_k(V_+)$ of the gaussian function as the distance between $x_{+,k}(V_+)$ and $x_{-,k}(V_+)$, i.e.

30 $$\begin{aligned} w_k(V_+)=x_{+,k}(V_+)-x_{-,k}(V_+). \end{aligned}$$

Using Eqs. (15) and (27), the condition (29) at either state value $x_{\mp ,k}(V_+) \in \{x_{-,k}(V_+),x_{+,k}(V_+)\}$ can be recast as

31 $$\begin{aligned} \exp \left( \alpha (x_{\mp ,k}(V_+),V_+)\right) =\frac{1}{k} \cdot \exp \left( \alpha (x_{max}(V_+),V_+)\right) . \end{aligned}$$

Employing Eqs. (16) and (28), defining

32 $$\begin{aligned} \beta (V_+) \triangleq \frac{V_+^2 \cdot x_{\text {on}}^2}{\sigma _{\text {p}}}, \end{aligned}$$

and recalling the earlier specified formula (18) for $\gamma (V_+)$, the constraint (31) reduces to the second-order polynomial

33 $$\begin{aligned} x_{\mp ,k}^2(V_+)-\beta (V_+) \cdot \gamma (V_+) \cdot x_{\mp ,k}(V_+)+\frac{1}{4} \cdot \beta ^2(V_+) \cdot \gamma ^2(V_+)-x^2_{\text {on}} \cdot \ln (k)=0, \end{aligned}$$

which can be easily solved for $x_{\mp ,k}(V_+)$, yielding

34 $$\begin{aligned} x_{\mp ,k}(V_+)=\frac{1}{2} \cdot \left( \beta (V_+) \cdot \gamma (V_+) \mp 2 \cdot x_{\text {on}} \cdot \sqrt{\ln (k)} \right) . \end{aligned}$$

Inserting (34) into Eq. (30), the $k$th-scale width $w_k(V_+)$ of the gaussian function $g_{\text {SET}}(x,V_+)$ is then computable via

35 $$\begin{aligned} w_k(V_+)=w_k=2 \cdot x_{\text {on}} \sqrt{\ln (k)}, \end{aligned}$$

which, as indicated, is surprisingly found to be independent of $V_+$. Fig. 9f depicts the *k*th-scale width $w_k(V_+)$ of the gaussian bell-shaped SET state evolution function $g_{\text {SET}}(x,V_+)$ against $V_+$ for the first, second, and third *k* value from the set $\{1.5,2,3\}$, as computed through the exact analytical formula (35), delivering in turn the constant values 0.076, 0.100, and 0.126 (red, magenta, and green dashed traces, respectively) as well as by numerical means (blue, black, and brown solid traces, respectively).

### A systematic methodology to craft the pulse stimulus for enabling the ReRAM cell to support multiple oscillations around prescribed resistance levels

Having acquired key geometrical properties of a gaussian bell-shaped SET state evolution function $g_{\text {SET}}(x,V)$, and, particularly, the positive value $V_+$ to be assigned to the DC voltage *V* to program its peak level at a prescribed state value $x_{\text {max}}$, as reported in section “Positive DC voltage for programming the abscissa of the peak of a SET SDR”, and its *k*th-scale width $w_k$, as described in section “Width of the Gaussian bell-shaped SET state evolution function”, we are now in a position to present a systematic technique for choosing $2\cdot P-1$ parameters of a generalised pulse train from the class illustrated in Fig. 1c, specifically $V_{+,1}, V_{+,2}, \dots , V_{+,P}, \tau _{+,1}, \tau _{+,2}, \ldots , \tau _{+,P}, \tau _-$, with $V_{+,1}<V_{+,2}<\ldots <V_{+,P}$, so as to induce the coexistence of a predefined number of asymptotic oscillatory solutions for the memory state of the ReRAM cell around prescribed levels ($P\in \mathbb {N}_{>0}$), for a given RESET pulse height $V_-$, chosen beforehand.

Our methodology envisages to endow the TA-SE (10) with as many stable equilibria as the number *P* of positive pulses over each cycle of the pulse train voltage signal, falling across the ReRAM cell. In particular, the proposed systematic procedure is calibrated so as to ensure that the graph of the leftmost scaled gaussian bell-shaped state evolution function $\tilde{\tau }_{+,1} \cdot g_{\text {SET}}(\bar{x},V_{+,1})$, corresponding to the first positive input pulse, which features the smallest height $V_{+,1}$, creates one and only one stable TA-SE equilibrium $\bar{x}_{\text {eq},1}$ with the graph of the modulus of the scaled RESET state evolution function $\tilde{\tau }_{-} \cdot g_{\text {RESET}}(\bar{x},V_{-})$, appearing on the right hand side of Eq. (12). It also guarantees that the graph of the scaled gaussian bell-shaped state evolution function $\tilde{\tau }_{+,j} \cdot g_{\text {SET}}(\bar{x},V_{+,j})$, corresponding to the $j$th positive input pulse, which exhibits height $V_{+,j}$, forms a pair of TA-SE equilibria, specifically $\bar{x}_{\text {eq},2 \cdot j-2}$ and $\bar{x}_{\text {eq},2 \cdot j-1}$, featuring an unstable and stable nature, respectively, with the graph of $\tilde{\tau }_{-} \cdot |g_{\text {RESET}}(\bar{x},V_{-})|$, for $j\in \{2,\ldots ,P\}$. The target of the methodology is to massage the aforementioned $2\cdot P-1$ parameters of the generalised pulse train so as to endow the resulting TA-SE (10) with $2 \cdot P-1$ equilibria, indicated as $\bar{x}_{\text {eq},1}$, $\bar{x}_{\text {eq},2}$, $\bar{x}_{\text {eq},2 \cdot P-1}$.

According to the TA-SDR analysis, the stable ones, endowed with odd labels, and referred to as $\bar{x}_{\text {eq},1}$, $\bar{x}_{\text {eq},3}$, $\ldots$, $\bar{x}_{\text {eq},2 \cdot P-1}$, are expected to denote the mean values of the *P* admissible stable oscillatory solutions $x_{1,ss}(t)$, $x_{3,ss}(t)$, $\dots$, and $x_{2 \cdot P-1,ss}(t)$ for the memory state *x* of the periodically-forced ReRAM cell.

In order for the *i*th scaled SET state evolution function, with $i\in \{1,2,\cdot ,P\}$, to dominate over the other $P-1$ terms in the sum, composing the SET TA-SE component $\dot{\bar{x}}_{\text {SET}}$ from Eq. (14), locally, around the respective maximum, which is a necessary critical measure to ensure that the existence of TA-SE equilibria in the region around $x_{\text {max},i}$ is determined mainly by the interaction between the $\tilde{\tau }_{+,i} \cdot g_{\text {SET}}(\bar{x},V_{+,i})$ and the $\tilde{\tau }_{-} \cdot |g_{\text {RESET}}(\bar{x},V_{-})|$ versus $\bar{x}$ loci, the *P* stable equilibria to be provided as a design specification to the input of the systematic procedure need to hold a suitable distance, which is at least one *k*th-scale width $w_k$, one from any adjacent other. Moreover, for each *i* value from the set $\{1,2,\dots ,P\}$, the abscissa $x_{\text {max},i}$ of the maximum of the gaussian SET state evolution function $g_{\text {SET}}(\bar{x},V)$, sampled at the DC voltage $V_{+,i}$, which corresponds to the height of the *i*th positive pulse within each period of the train stimulus, is placed to the left of the prescribed $(2 \cdot i-1)$th stable TA-SE equilibrium $\bar{x}_{eq,2 \cdot i-1}$, at an appropriate distance, amounting to one quarter of the *k*th-scale bell width $w_k$, from its location. This step ensures that for each $i\in \{1,2,\dots ,P\}$ the SET (RESET) forces win over the RESET (SET) ones to the left (right) of the $(2 \cdot i-1)$th TA-SE equilibrium $\bar{x}_{eq,2 \cdot i-1}$, which, as a result, acquires a stability nature, as explained in section “The time average state dynamic route technique”. Having computed the state value, at which the peak of the *i*th gaussian bell should appear, via $x_{\text {max},i}=\bar{x}_{\text {eq},2 \cdot i-1}-w_k/4$, for $i\in \{1,2,\ldots ,P\}$, the approximate closed-form expression (22), with $x_{\text {max}}=x_{\text {max},i}$, $V_{+,1}=V^{(\text {opt})}_{+,1}$, and $V_{+,2}=V^{(\text {opt})}_{+,2}$, is then employed to compute the positive value $V_{+,i}$ to be assigned to the DC voltage *V* in the expression for the SET state evolution function $g_{\text {SET}}(\bar{x},V)$, appearing in the *i*th addend of the sum to the right hand side of Eq. (14), i.e. the height of the *i*th positive pulse over each cycle of the train excitation signal $v_S$. *P* algebraic equations are then written down to enforce the TA-SE (10) to feature equilibria at $\bar{x}_{eq,1}$, $\bar{x}_{eq,3}$, $\ldots$, and $\bar{x}_{eq,2 \cdot P-1}$. More specifically, these constraints, imposing an equality between the moduli of the SET $\dot{\bar{x}}_{\text {SET}}$ and RESET $\dot{\bar{x}}_{\text {RESET}}$ TA-SE components at each of the stable equilibria, which Eq. (10) is expected to admit, read as

36 $$\begin{aligned}{} & {} r_{+,1} \cdot g_{\text {SET}}(\bar{x}_{eq,1},V_{+,1})+r_{+,2} \cdot g_{\text {SET}}(\bar{x}_{eq,1},V_{+,2})+\ldots +r_{+,P} \cdot g_{\text {SET}}(\bar{x}_{eq,1},V_{+,P})=-g_{\text {RESET}}(\bar{x}_{eq,1},V_{-,1}), \nonumber \\ \end{aligned}$$

37 $$\begin{aligned}{} & {} r_{+,1} \cdot g_{\text {SET}}(\bar{x}_{eq,3},V_{+,1})+r_{+,2} \cdot g_{\text {SET}}(\bar{x}_{eq,3},V_{+,2})+\ldots +r_{+,P} \cdot g_{\text {SET}}(\bar{x}_{eq,3},V_{+,P})=-g_{\text {RESET}}(\bar{x}_{eq,2},V_{-,1}), \ \text {and} \nonumber \\ \end{aligned}$$

38 $$\begin{aligned}{} & {} \ldots \nonumber \\{} & {} r_{+,1} \cdot g_{\text {SET}}(\bar{x}_{eq,2 \cdot P-1},V_{+,1})+r_{+,2} \cdot g_{\text {SET}}(\bar{x}_{eq,2 \cdot P-1},V_{+,2})+\ldots +r_{+,P} \cdot g_{\text {SET}}(\bar{x}_{eq,2 \cdot P-1},V_{+,P})\nonumber \\{} & {} \quad =-g_{\text {RESET}}(\bar{x}_{eq,2 \cdot P-1},V_{-,1}), \end{aligned}$$

where $r_{+,1} \triangleq \tau _{+,1}/\tau _{-}$, $r_{+,2} \triangleq \tau _{+,2}/\tau _{-}$, $\dots$, and $r_{+,P} \triangleq \tau _{+,P}/\tau _{-}$ express the first, second, $\dots$, and *P*th SET to RESET pulse width ratio, respectively. This set of *P* equations is then solved for the unknowns $r_{+,1}$, $r_{+,2}$, $\ldots$, and $r_{+,P}$. The last unknown parameter, specifically the RESET pulse width $\tau _{-}$, which automatically fixes all the SET pulse widths $\tau _{+,1}$, $\tau _{+,2}$, $\ldots$, and $\tau _{+,P}$, is finally chosen so small as to guarantee the accuracy of the predictions, drawn from the TA-SDR analysis, as verifiable through the investigation of the SCPCM of the periodically-forced memristive system, as well as via numerical simulations.

#### Remark 2

Even though an adequate distance between adjacent TA-SE equilibria is preliminarily observed in their prescription, as specified above, for an arbitrary choice of the negative input pulse height $V_-$, out of the theoretic methodology, presented in this section, the leftmost scaled gaussian bell-shaped $\tilde{\tau }_{+,1} \cdot g_{\text {SET}}(\bar{x},V_{+,1})$ versus $\bar{x}$ locus may cross the graph of $\tilde{\tau }_{-} \cdot |g_{\text {SET}}(\bar{x},V_{-})|$ as a function of $\bar{x}$ a couple of additional times, for some choices of the height $V_{+,1}$ of the first positive pulse and of the first SET-to-RESET pulse width ratio $r_{+,1}$. However, ad hoc control measures can be set in place to ensure that the interaction between the first scaled SET SDR and the modulus of the only scaled RESET SDR forms one and only one GAS equilibrium at the prescribed location $\bar{x}_{\text {eq},1}$ for Eq. (10). For example, with reference to the numerical study, discussed in section “On the crossings between one scaled SET SDR and one scaled RESET SDR”, and referring to the simple case, where a two-pulse-per-cycle pulse train stimulus is let fall across the ReRAM cell, choosing $V_-=-0.5 \, \text{V}$, the resulting TA-SE equation admits one and only one GAS equilibrium $\bar{x}_{\text {eq}}$, irrespective of the pulse width ratio *r*, if $V_+$ is set to a value lower than the abscissa $\hat{V}_+=0.494 \, \text{V}$ of the cusp in Fig. 8a. The state value $x_{\text {max}}$, at which the $g_{\text {SET}}(\bar{x},V_+)$ versus $\bar{x}$ locus features a peak for $V_+=\hat{V}_+$ is 0.266. This directly sets the maximum value, which may be prescribed for the TA-SE equilibrium $\bar{x}_{\text {eq}}$, so as to ensure its GAS property, irrespective of *r*, to $x_{\text {max}}+w_k/4$, that equals 0.285, 0.291, and 0.297, for the first, second, and third *k* value from the set $\{1.5,2,3\}$. In principle, as is the case for the examples illustrated in Figs. 13, 14, and 15, assuming a *P*-pulse-per-cycle pulse train voltage stimulus were let fall across the ReRAM cell, it is also possible to set the first TA-SE equilibrium $\bar{x}_{\text {eq},1}$ to a value larger than this upper bound, but then, after solving the system of linear Eqs. (36)–(38), it would be necessary to check that the selection of values for the first pulse height and for the first SET-to-RESET pulse width ratio would fall in the monostability green region of the coloured *r* versus $V_+$ map, with $r=r_{+,1}$, under the specified value for $V_-$. Finally, it is worth pointing out that a suitable change in the value, assigned to $V_-$, may allow to move the abscissa $\hat{V}_+$ of the cusp, indicating the left bound of the red bistability domain, to the right, relative to its location along the horizontal axis in the coloured map of Fig. 8a. With reference to the proposed methodology, this would result in a corresponding increase in the maximum value, which may be prescribed for the stable equilibrium $\bar{x}_{\text {eq},1}$, that the leftmost scaled gaussian bell-shaped SET SDR would form with the graph of the modulus of the scaled RESET state evolution function over the time average state, irrespective of the first SET-to-RESET pulse width ratio $r_{+,1}$.

#### Remark 3

**Figure 11:**
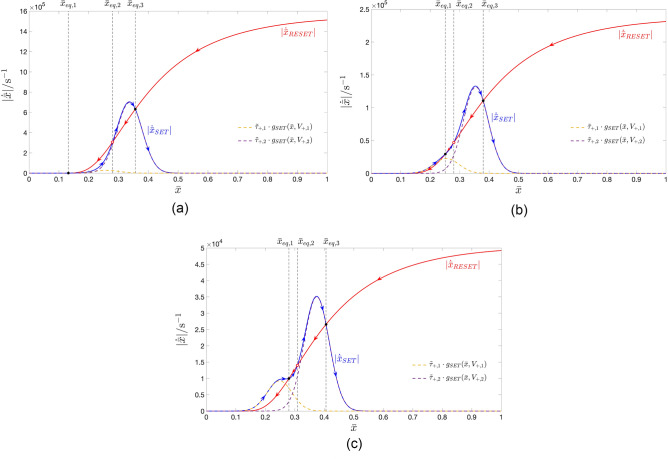
Illustrations elucidating how to choose the design parameter *k* for a case study, where it is requested for the ReRAM cell to act as a bistable oscillator under the application of a three-pulse-per-cycle pulse train voltage stimulus between its terminals. Let the *i*th positive pulse in the input sequence over each cycle have amplitude $V_{+,i}$ and width $\tau _{+,i}$, for $i\in \{1,2\}$. The negative pulse, following the two positive ones in each input cycle, is assumed to feature a fixed amplitude $V_-$ of $-0.5\text{V}$, while its width $\tau _-$ is to be determined. It is further required for the left LAS TA-SE equilibrium $\bar{x}_{\text {eq},1}$ to be located at 0.280. The right LAS TA-SE equilibrium $\bar{x}_{\text {eq},3}$ should be apart from the left one by one bell width $w_k$. When *k* is set to 1.5, 2, and 3, $\bar{x}_{\text {eq},3}$ is expected to lie at 0.356, 0.380, and 0.406, respectively. (**a**) For $k=1.5$ the application of the design methodology first employs the approximate analytical formula (22), with $x_{\text {max}}$ set to $x_{\text {max},1}=\bar{x}_{\text {eq},1}-w_{1.5}/4$ ($x_{\text {max},2}=\bar{x}_{\text {eq},3}-w_{1.5}/4$), $V_{+,1}=V^{(\text {opt})}_{+,1}$, and $V_{+,2}=V^{(\text {opt})}_{+,2}$, to fix the amplitude $V_{+,1}$ ($V_{+,2}$) of the first (second) SET pulse to $0.483 \, \text{V}$ ($0.550 \, \text{V}$). It then specifies the values 0.815 and $2.687 \times 10^{-5}$ for $r_{+,1}$ and $r_{+,2}$, respectively, by solving the linear system of equations (36)–(37). Regardless of the choice for the RESET pulse width $\tau _-$, which automatically fixes the values for the SET pulse widths $\tau _{+,1}$ and $\tau _{+,2}$, the TA-SE is found to admit the triplet of equilibria $(\bar{x}_{\text {eq},1},\bar{x}_{\text {eq},2},\bar{x}_{\text {eq},3})=(0.132,0.28,0.356)$. Clearly, the design specifications are not satisfied here. (**b**) For $k=2$, applying the proposed methodology delivers first the SET pulse heights $V_{+,1}=0.478 \, \text{V}$, and $V_{+,2}=0.564 \, \text{V}$, and then the SET-to-RESET pulse width ratios $r_{+,1}=10.866$ and $r_{+,2}=8.974 \times 10^{-7}$. The TA-SE equilibria are then found to lie at $\bar{x}_{\text {eq},1}=0.251$, $\bar{x}_{\text {eq},2}=0.28$, and $\bar{x}_{\text {eq},3}=0.38$. Also in this case the systematic procedure, introduced in this paper, fails to fulfil the design tasks. (**c**) Recurring to the proposed design methodology with $k=3$, the pulse train voltage stimulus is crafted as specified by the parameters $V_{+,1}=0.472 \, \text{V}$, $V_{+,2}=0.580 \, \text{V}$, $r_{+,1}=54.759$, and $r_{+,2}=1.715 \times 10^{-8}$. The TA-SE admits here the equilibria $\bar{x}_{\text {eq},1}=0.280$, $\bar{x}_{\text {eq},2}=0.309$, and $\bar{x}_{\text {eq},3}=0.406$. Therefore, choosing $k=3$, the combination between the two gaussian bells and the red curve, increasing monotonically with the time average state, allows to endow the TA-SE with two LAS equilibria at the desired locations, meeting the design requirements.

**Figure 12:**
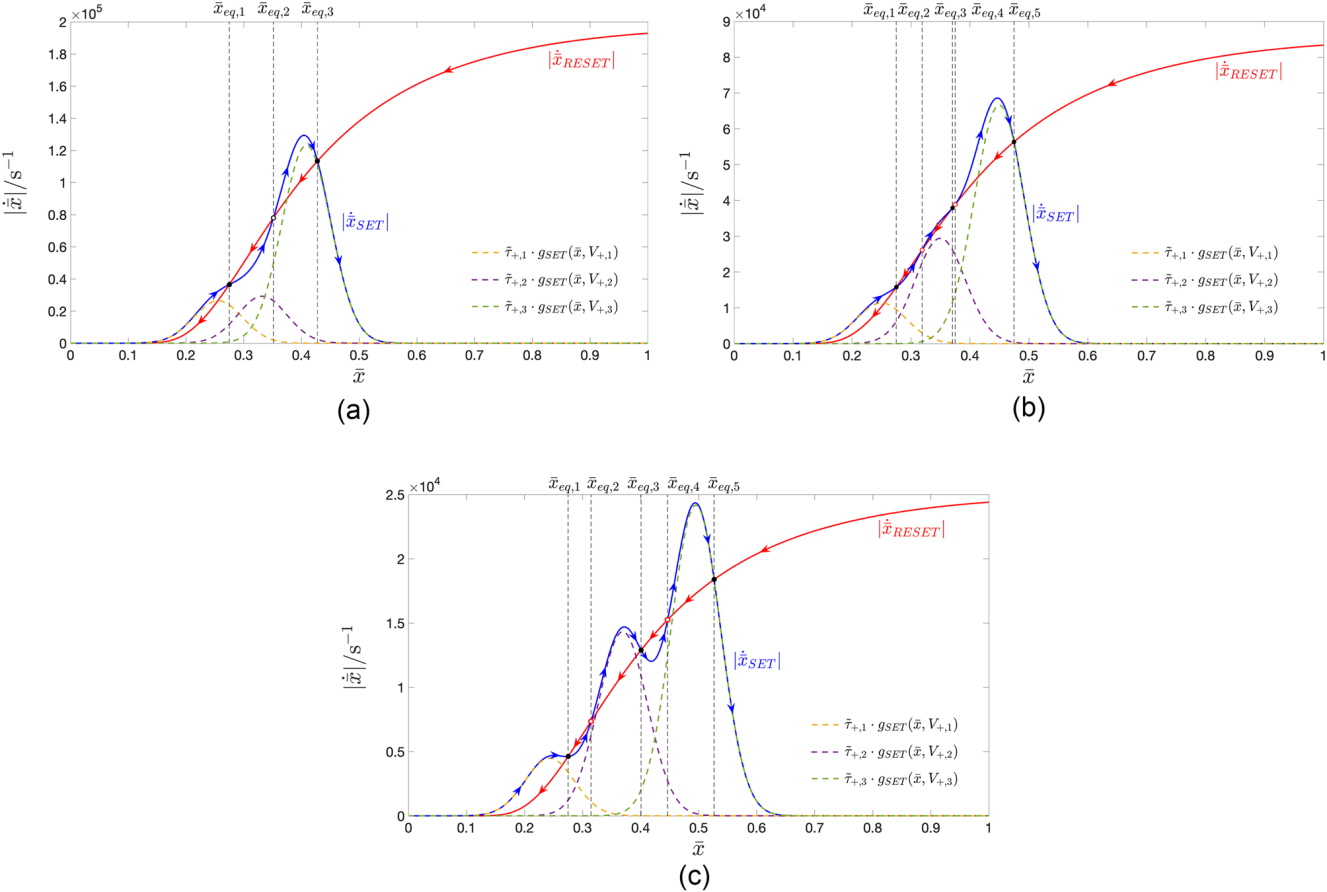
Graphs revealing the instrumental role of the TA-SDR analysis tool to guide the circuit designer toward an appropriate choice for the parameter *k* for a case study, where a pulse train voltage stimulus, composed of one negative and three positive pulses per cycle, is expected to induce tristability in the oscillatory response of the ReRAM cell. Let $V_{+,i}$ ($\tau _{+,i}$) indicate the pulse amplitude (width) of the $i$th SET pulse, for $i\in \{1,2,3\}$. The pulse amplitude $V_-$ of the RESET pulse is fixed to $-0.5 \, \text{V}$, while its width $\tau _-$ is an unknown variable. The leftmost LAS TA-SE equilibrium $\bar{x}_{eq,1}$ should lie at 0.275. The *j*th equilibrium $\bar{x}_{eq,j}$ should appear to the right of the $(j-1)$th equilibrium $\bar{x}_{eq,j-1}$ by as much as one bell width $w_k$, for $j\in \{2,3\}$. For *k* equal to 1.5, 2, and 3, the inner (rightmost) LAS TA-SE equilibrium $\bar{x}_{\text {eq},3}$ ($\bar{x}_{\text {eq},5}$) is expected to lie at 0.351 (0.428), 0.375 (0.475), and 0.401 (0.527), respectively. (**a**) Choosing $k=1.5$, the proposed systematic design procedure first specifies the values $0.478 \, \text{V}$, $0.546 \, \text{V}$, and $0.606 \, \text{V}$ for the SET pulse amplitudes $V_{+,1}$, $V_{+,2}$, and $V_{+,3}$, respectively, via the approximate analytical formula (22), for $V_{+,1}=V^{(\text {opt})}_{+,1}$, and $V_{+,2}=V^{(\text {opt})}_{+,2}$, and fixing $x_{\text {max}}$ in turn to $x_{\text {max},1}=\bar{x}_{\text {eq},1}-w_{1.5}/4$, $x_{\text {max},2}=\bar{x}_{\text {eq},3}-w_{1.5}/4$, and $x_{\text {max},3}=\bar{x}_{\text {eq},5}-w_{1.5}/4$. It then solves the system of linear Eqs. (36)–(38) with $P=3$ for $r_{+,1}$, $r_{+,2}$, and $r_{+,3}$, in turn found to equal 13.228, $2.375 \times 10^{-5}$, and $5.399 \times 10^{-12}$. Irrespective of the choice for $\tau _-$, which directly sets values for $\tau _{+,i}$, with $i\in \{1,2,3\}$, the intersections between the loci of the moduli of the SET and RESET TA-SE components, identifying the equilibria $\bar{x}_{eq,1}$, $\bar{x}_{eq,2}$, and $\bar{x}_{eq,3}$, the outer (the inner) of which are LAS (is unstable), for Eq. (10), are found to lie at 0.275, 0.351, and 0.428, respectively. As the TA-SDR analysis predicts bistability in the memristor steady-state oscillatory behaviour, assigning 1.5 to *k* is not an appropriate design choice. (**b**) For $k=2$, out of the proposed design procedure, the input parameters $V_{+,1}$, $V_{+,2}$, $V_{+,3}$, $r_{+,1}$, $r_{+,2}$, and $r_{+,3}$, are respectively set to $0.473 \, \text{V}$, $0.560 \, \text{V}$, $0.636 \, \text{V}$, 31.913, $1.578 \times 10^{-6}$, and $2.016 \times 10^{-16}$. Correspondingly, the TA-SE admits the five equilibria $\bar{x}_{eq,1}=0.275$, $\bar{x}_{eq,2}=0.319$, and $\bar{x}_{eq,3}=0.370$, $\bar{x}_{eq,4}=0.375$, and $\bar{x}_{eq,5}=0.475$, of which those labelled with odd numbers are LAS. Here the systematic parameter tuning procedure meets the design specifications. However the robustness of the design is questionable, given the non-ideal proximity between the TA-SE equilibria $\bar{x}_{\text {eq},3}$ and $\bar{x}_{\text {eq},4}$. (**c**) With $k=3$, the application of the design procedure allows to choose the input parameters $V_{+,1}=0.467 \, \text{V}$, $V_{+,2}=0.576 \, \text{V}$, $V_{+,3}=0.668 \, \text{V}$, $r_{+,1}=1.115\times 10^{2}$, $r_{+,2}=4.240 \times 10^{-8}$, and $r_{+,3}=8.234\times 10^{-22}$. The $|\dot{\bar{x}}_{\text {SET}}|$ versus $\bar{x}$ and $|\dot{\bar{x}}_{\text {RESET}}|$ versus $\bar{x}$ loci feature the five crossings $\bar{x}_{eq,1}=0.275$, $\bar{x}_{eq,2}=0.315$, and $\bar{x}_{eq,3}=0.401$, $\bar{x}_{eq,4}=0.446$, and $\bar{x}_{eq,5}=0.527$. Those, labelled with odd numbers, are LAS TA-SE equilibria, as desired.

The selection of the real-valued parameter *k* is a critical design choice. In order to gain insights into this important aspect, Figs. 11 and 12 illustrate two examples, where the methodological approach, presented in this section, is applied for different *k* values in the attempt to endow the TA-SE with two or three prescribed equilibria, respectively. In each of the two figures, plots (a), (b), and (c) show the loci of the moduli of the SET and RESET components of the corresponding TA-SE for the first, second, and third *k* value in the set $\{1.5,2,3\}$, revealing how only assigning the largest value in this triplet to the parameter under discussion allows to satisfy the design specifications (see the respective captions for more detail).

Importantly, under a proper selection for *k*, constraining the SET and RESET TA-SE components to comply with the set of *P* constraints (36)–(38), together with the imposition of a minimum distance between adjacent stable equilibria, prescribed for the TA-SE, as well as with a sufficient leftward shift of each SET SDR relative to the respective stable TA-SE equilibrium, the scaled gaussian bells, resulting from the application of the proposed algorithm, gracefully pass over the locus of the modulus of the scaled RESET state evolution function versus the time average state in the regions of the respective peaks only, as may be inferred from either of Figs. 11c and 12c, which refer to a particular case study for $P=2$ and for $P=3$, respectively, and where, as a result, the blue-coloured single-valued curve, illustrating the TA-SE component, is found to oscillate around the graph of the modulus of the RESET TA-SE component as a function of the time average state, creating $2\cdot P-1$ equilibria, of which *P* stable, as prescribed, for the TA-SE. In each of the case studies, illustrating the application of the theory in section 6, keeping such a value for *k*, which implies a minimum distance between adjacent prescribed stable TA-SE equilibria of $w_3=0.126$, and a spacing between each prescribed stable TA-SE equilibrium and the abscissa of the peak of the respective gaussian bell of $w_3/4=0.031$, proves to be a suitable choice to accomplish a robust design.

## Discussion

The first part of this section applies the rigorous system-theoretic methodology, presented in section “A systematic methodology to craft the pulse stimulus for enabling the ReRAM cell to support multiple oscillations around prescribed resistance levels”, to the Strachan model^4^ for the determination of heights and widths of all the pulses, appearing cyclically across the ReRAM cell, so as to endow it with three, four, or five coexisting oscillatory operating modes around prescribed resistance levels. The second part of this section is devoted to show an interesting potential application, where the local fading memory effects, emerging across the nonvolatile resistance switching memory under periodic pulse train stimulation, could be leveraged to counteract certain non-idealities, which may be responsible for the corruption of the synaptic weights stored in a crossbar array.

### Application of the theory to endow the ReRAM cell with three, four, or five oscillatory behaviours

**Figure 13:**
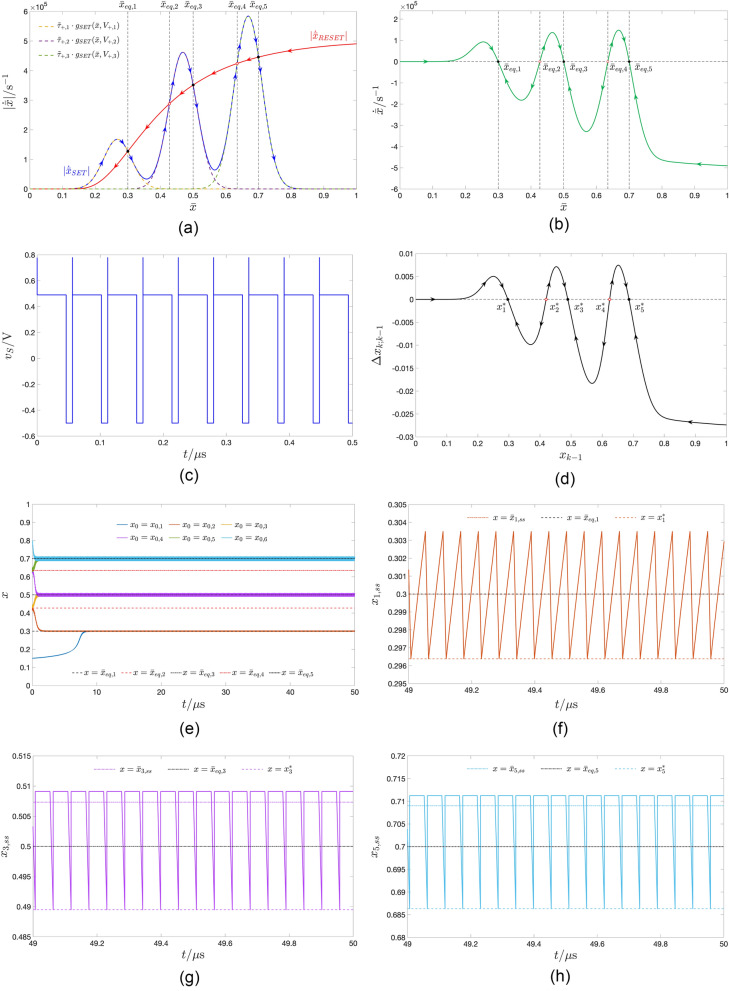
(**a**) Decomposition of the TA-SDR into its SET (blue trace) and RESET (red trace) contributions, here plotted together on the $|\dot{\bar{x}}|$ versus $\bar{x}$ plane to visualise each possible equilibrium $\bar{x}_{\text {eq}}$ of equation (10), where $\dot{\bar{x}}_{\text {SET}} = -\dot{\bar{x}}_{\text {RESET}}$, for a case study, where the proposed methodology from section 5.3 set the values for the parameters $V_{+,1}$, $V_{+,2}$, $V_{+,3}$, $r_{+,1}$, $r_{+,2}$, and $r_{+,3}$ of a four-pulse-per-cycle pulse train voltage stimulus, with $V_{-}$ preliminarily chosen as $-0.5\,\text{V}$, to $+0.490\,\text{V}$, $+0.649\,\text{V}$, $+0.778\,\text{V}$, 4.594, $1.489 \times 10^{-18}$, and $1.361 \times 10^{-47}$, respectively, so as to endow the TA-SE with the 3 stable equilibria $\bar{x}_{\text {eq},1}=0.3$, $\bar{x}_{\text {eq},3}=0.5$, and $\bar{x}_{\text {eq},7}=0.7$, which in turn place the maxima of the first, second, and third gaussian bells at $x_{\text {max},1}=0.269$, $x_{\text {max},2}=0.469$, and $x_{\text {max},3}=0.669$. The TA-SE equilibria are found to lie at $\bar{x}_{eq,1}=0.3$, $\bar{x}_{eq,2}=0.427$, $\bar{x}_{eq,3}=0.5$, $\bar{x}_{eq,4}=0.635$, and $\bar{x}_{eq,5}=0.7$, the odd numbered of which are LAS, as requested. (**b**) TA-SDR of the ReRAM cell under a voltage excitation signal from the class identified by the aforegiven parameter set of cardinality 7. (**c**) Time waveform of a particular generalised pulse train voltage stimulus $v_S$, extracted from the class under focus by setting $\tau _-$ to $1 \times 10^{-8}\,\text{s}$, which directly fixes $\tau _{+,1}$, $\tau _{+,2}$, and $\tau _{+,3}$ to $4.594 \times 10^{-8} \,\text{s}$, $1.489 \times 10^{-26} \,\text{s}$, and $1.361 \times 10^{-55} \,\text{s}$, respectively. (**d**) SCPCM of the ReRAM cell, subject to the specific generalised pulse train from (**c**), confirming the predictions drawn from the TA-SDR analysis. (**e**) Transients in the memory state *x* of the ReRAM cell, as resulting from numerical simulations of the Strachan model, with *v* taken identically equal to the excitation signal $v_S$ from (**c**), for each initial condition $x_0$ from the set $\{x_{0,1}, x_{0,2}, x_{0,3}, x_{0,4}, x_{0,5}, x_{0,6}\}=\{0.15,0.415,0.425,0.620,0.626,0.8\}$. From either of the first two, of the second two, and of the last two initial conditions the memory state of the ReRAM cell asymptotically approaches the steady-state oscillatory solutions $x_{\text {ss},1}$, $x_{\text {ss},3}$, and $x_{\text {ss},5}$, which revolve approximately around $\bar{x}_{\text {eq},1}$, $\bar{x}_{\text {eq},3}$, and $\bar{x}_{\text {eq},5}$, respectively, as illustrated in turn in plots (**f**), (**g**), and (**h**), visualising also their time averages $\bar{x}_{\text {ss},1}$, $\bar{x}_{\text {ss},3}$, and $\bar{x}_{\text {ss},5}$, and the corresponding stable map fixed points $x^{*}_1$, $x^{*}_3$, and $x^{*}_5$.

**Figure 14:**
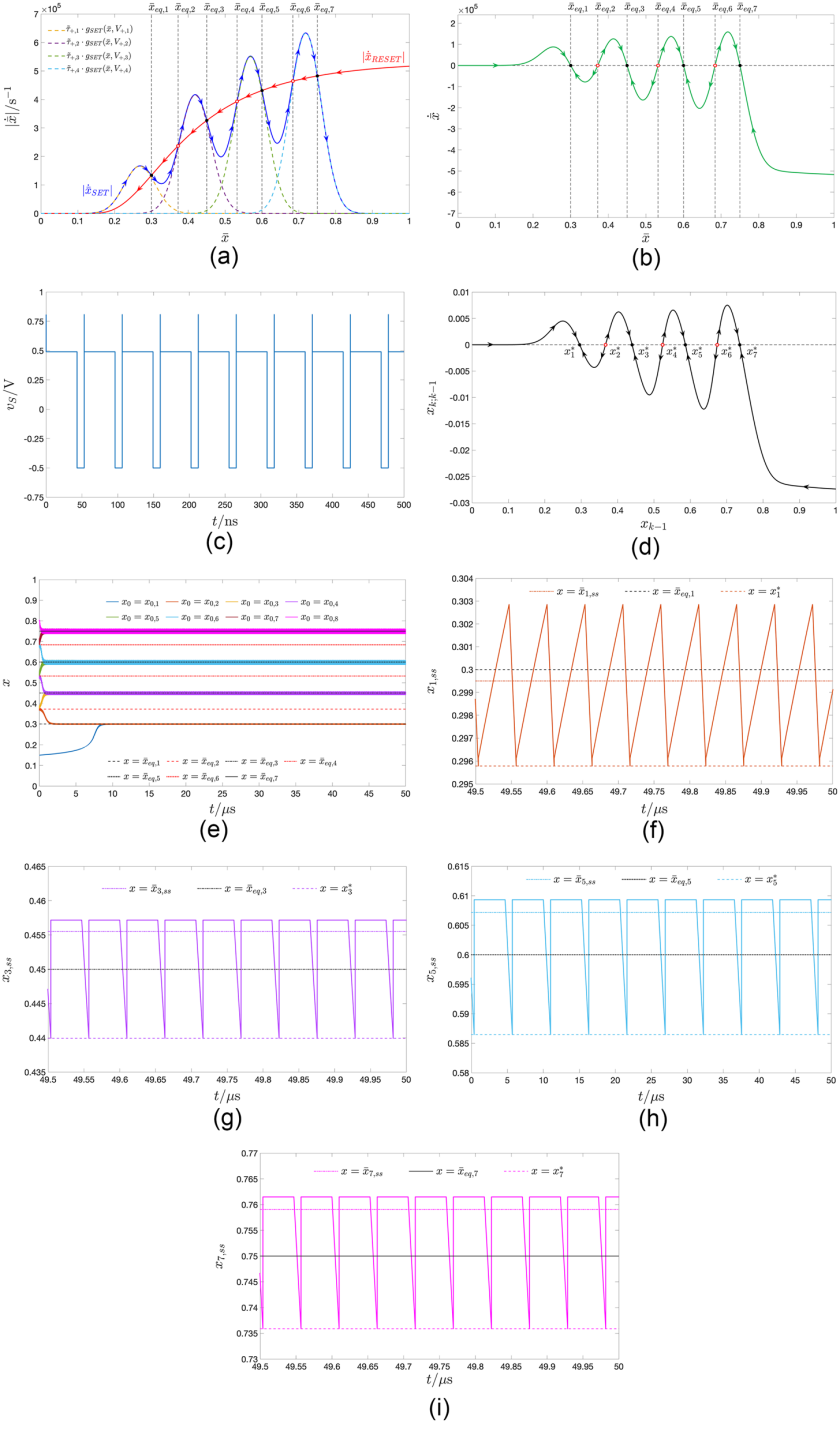
(**a**) Blue (Red) trace: $|\dot{\bar{x}}_{\text {SET}}|$ $(|\dot{\bar{x}}_{\text {RESET}}|$ versus $\bar{x}$ locus resulting from the application of the theoretical methodology from section 5.3 to the Strachan model so as to endow the TA-SE with 4 stable equilibria $\bar{x}_{\text {eq},1}$, $\bar{x}_{\text {eq},3}$, $\bar{x}_{\text {eq},5}$, and $\bar{x}_{\text {eq},7}$ at 0.3, 0.45, 0.6, and 0.75, respectively. First the maxima $x_{\text {max},1}$, $x_{\text {max},2}$, $x_{\text {max},3}$, and $x_{\text {max},4}$ of the four gaussian bells were in turn positioned at 0.269, 0.419, 0.569, and 0.719. The amplitudes $V_{+,1}$, $V_{+,2}$, $V_{+,3}$, and $V_{+,4}$ of the four positive pulses were then chosen as $0.490 \, \text{V}$, $0.613 \, \text{V}$, $0.717 \, \text{V}$, and $0.807 \, \text{V}$, while $V_-$ was preliminarily fixed to $-0.5 \, \text{V}$. Finally, the first, second, third, and fourth pulse width ratios $r_{+,1}$, $r_{+,2}$, $r_{+,3}$, and $r_{+,4}$ were respectively taken as 4.312, $6.162 \times 10^{-13}$, $8.667 \times 10^{-32}$, and $1.802 \times 10^{-56}$. The TA-SE admits equilibria at $\bar{x}_{\text {eq},1}=0.3$, $\bar{x}_{\text {eq},2}=0.372$, $\bar{x}_{\text {eq},3}=0.45$, $\bar{x}_{\text {eq},4}=0.532$, $\bar{x}_{\text {eq},5}=0.6$, $\bar{x}_{\text {eq},6}=0.684$, $\bar{x}_{\text {eq},7}=0.75$, of which those labeled with odd numbers are LAS, as desired. (**b**) TA-SDR of the ReRAM cell subject to any input train featuring the aforementioned 9 parameters. (**c**) Time course of a particular pulse train extracted from the class by fixing the RESET pulse width $\tau _-$ to $1 \times 10^{-8} \, \text{s}$, which automatically sets the the widths $\tau _{+,1}$, $\tau _{+,2}$, $\tau _{+,3}$, and $\tau _{+,4}$ of the four SET pulses to $4.312 \times 10^{-8} \, \text{s}$, $6.162 \times 10^{-21} \, \text{s}$, $8.667 \cdot 10^{-40} \, \text{s}$, and $1.802 \times 10^{-64} \, \text{s}$, respectively. (**d**) SCPCM of the ReRAM cell, when its voltage *v* is forced to follow the generalised pulse train stimulus $v_S$ from (**c**) at all times, validating the TA-SDR analysis. (**e**) Solution to the ODE (1), with state evolution function (3), under the periodic stimulus from (**c**), and for each initial condition $x_0$ from the set $\{x_{0,1},x_{0,2},x_{0,3},x_{0,4},x_{0,5},x_{0,6},x_{0,7},x_{0,8}\}=\{0.15,0.365,0.375,0.52,0.535,0.67,0.685,0.8\}$. As may be evinced by monitoring the time course of the respective traces, the first, second, third, and fourth pair of initial conditions from this set respectively lie in the basins of attraction of the asymptotic memory state solutions $x_{1,\text {ss}}$, $x_{3,\text {ss}}$, $x_{5,\text {ss}}$, and $x_{7,\text {ss}}$, which in turn oscillate about the stable TA-SE equilibria $\bar{x}_{\text {eq},1}$, $\bar{x}_{\text {eq},3}$, $\bar{x}_{\text {eq},5}$, and $\bar{x}_{\text {eq},7}$, as may be inferred by inspecting plots (**f**), (**g**), (**h**), and (**i**), which also report their mean values $\bar{x}_{1,\text {ss}}$, $\bar{x}_{3,\text {ss}}$, $\bar{x}_{5,\text {ss}}$, and $\bar{x}_{7,\text {ss}}$, and the associated stable map fixed points $x^*_{1}$, $x^*_{3}$, $x^*_{5}$, and $x^*_{7}$.

**Figure 15:**
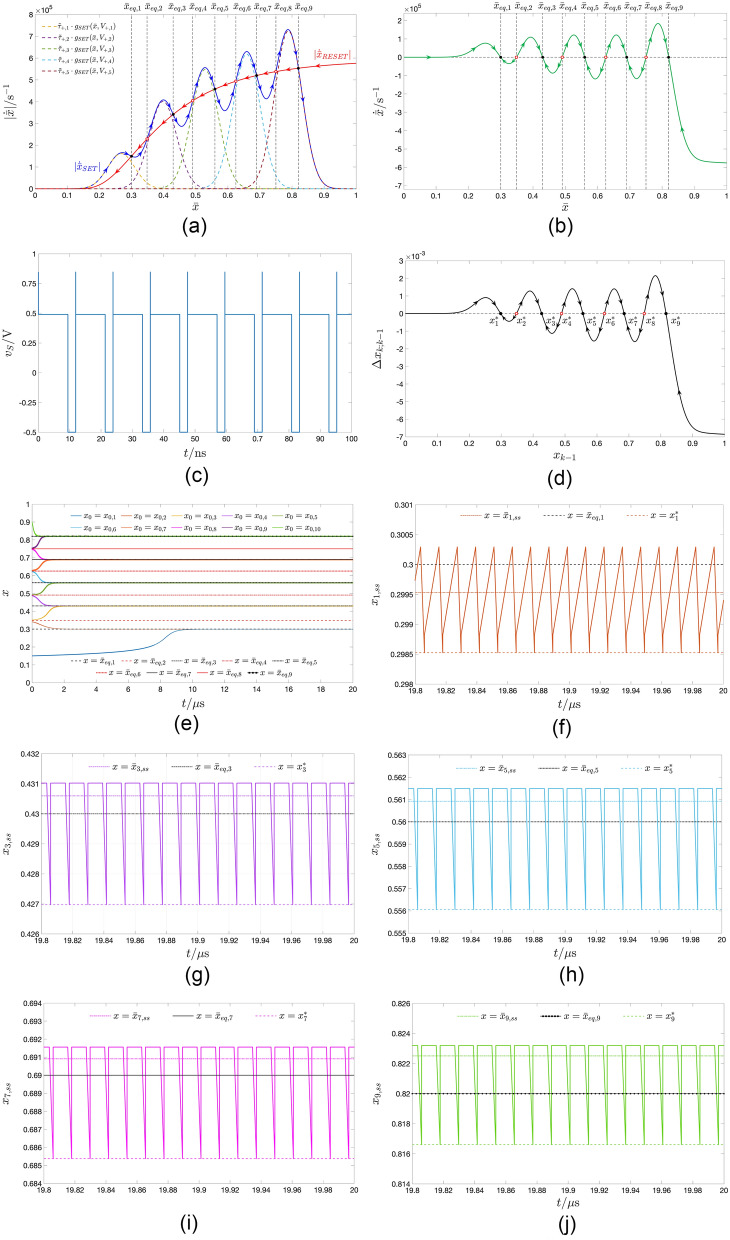
Moduli of the scaled SET (blue trace) and RESET (red trace) components of the TA-SE of the ReRAM cell subject to a six-pulse-per-cycle pulse train voltage stimulus from a class identified via the 11 parameters $V_{+,1}=0.490 \, \text{V}$, $V_{+,2}=0.598 \, \text{V}$, $V_{+,3}=0.690 \, \text{V}$, $V_{+,4}=0.772 \, \text{V}$, $V_{+,5}=0.847 \, \text{V}$, $r_{+,1}=3.764$, $r_{+,2}=6.651 \times 10^{-11}$, $r_{+,3}=3.241 \times 10^{-26}$, $r_{+,4}=6.156 \times 10^{-46}$, and $r_{+,5}=5.530 \times 10^{-70}$, and $V_-=-0.5 \, \text{V}$. While the last one was preliminarily chosen, the first 10 parameters were automatically determined via the analytical procedure outlined in section 5.3 so as to ensure Eq. (10) admits the 5 stable equilibria $\bar{x}_{\text {eq,1}}=0.3$, $\bar{x}_{\text {eq,3}}=0.43$, $\bar{x}_{\text {eq,5}}=0.56$, $\bar{x}_{\text {eq,7}}=0.69$, and $\bar{x}_{\text {eq,9}}=0.82$, which consequently fixed the maxima of the positive gaussian bells at $x_{\text {max},1}=0.269$, $x_{\text {max},2}=0.399$, $x_{\text {max},3}=0.529$, $x_{\text {max},4}=0.659$, and $x_{\text {max},5}=0.789$. The TA-SE is found to feature 5 LAS equilibria at the earlier prescribed locations, and unstable ones at $\bar{x}_{\text {eq,2}}=0.349$, $\bar{x}_{\text {eq,4}}=0.491$, $\bar{x}_{\text {eq,6}}=0.625$, and $\bar{x}_{\text {eq,8}}=0.750$. (**b**) TA-SDR of the ReRAM cell under a periodic pulse train from the above defined class. (**c**) Time waveform of a particular voltage excitation signal $v_S$, extracted from the aforementioned class by fixing the RESET pulse width $\tau _-$ to $2.5 \times 10^{-9} \, \text{s}$, which automatically set the widths of the five SET pulses $\tau _{+,1}$, $\tau _{+,2}$, $\tau _{+,3}$, $\tau _{+,4}$, and $\tau _{+,5}$ to $9.410 \times 10^{-9} \, \text{s}$, $1.663 \times 10^{-19} \, \text{s}$, $8.103 \times 10^{-35} \, \text{s}$, $1.539 \times 10^{-54} \, \text{s}$, and $1.383 \times 10^{-78} \, \text{s}$, respectively. (**d**) SCPCM of the ReRAM cell subject to the particular generalised pulse train voltage stimulus from (**c**), confirming the predictive capability of the TA-SDR analysis tool. (**e**) Time evolution of the memory state *x* of the ReRAM cell, as observed in numerical simulations of the Strachan model, where *v* was constrained to follow the voltage stimulus $v_S$ from (**c**) at all times, and for each initial condition $x_0$ from the set $\{x_{0,1},x_{0,2},x_{0,3},x_{0,4},x_{0,5},x_{0,6},x_{0,7},x_{0,8},x_{0,9},x_{0,10}\}=\{0.15,0.34,0.35,0.485,0.49,0.62,0.625,0.745,0.75,0.9\}$. When initiated from either initial condition in the first, second, third, fourth, and fifth pair, the memristor state *x* converges progressively toward the steady-state waveforms $x_{\text {ss},1}$, $x_{\text {ss},3}$, $x_{\text {ss},5}$, $x_{\text {ss},7}$, and $x_{\text {ss},9}$, respectively, as illustrated in plots (**f**), (**g**), (**h**), (**i**), and (**l**), which further visualise in turn the mean values $\bar{x}_{1,\text {ss}}$, $\bar{x}_{3,\text {ss}}$, $\bar{x}_{5,\text {ss}}$, $\bar{x}_{7,\text {ss}}$, and $\bar{x}_{9,\text {ss}}$ of the asymptotic oscillations, together with the corresponding stable map fixed points $x^*_{1}$, $x^*_{3}$, $x^*_{5}$, $x^*_{7}$, and $x^*_{9}$.

The first, second, and third examples, illustrated in turn in Figs. 13, 14, and 15, result from the application of the theoretical method, presented in section “A systematic methodology to craft the pulse stimulus for enabling the ReRAM cell to support multiple oscillations around prescribed resistance levels”, to the Strachan model^4^ for the specification of suitable values for the $2\cdot P-1$ tuneable parameters of a generalised pulse train voltage stimulus, belonging to the class, visualised in Fig. 1c, namely $V_{+,1}, V_{+,2}, \ldots , V_{+,P}, \tau _{+,1}, \tau _{+,2}, \ldots , \tau _{+,P}, \tau _-$, with $V_{+,1}<V_{+,2}<\ldots <V_{+,P}$, when $V_-$ is preliminarily set to $-0.5 \, \text{V}$, so as to induce the coexistence of *P* stable asymptotic oscillations with prescribed mean values $\bar{x}_{\text {eq},1}, \bar{x}_{\text {eq},3}, \dots , \bar{x}_{\text {eq},2 \cdot P-1}$ in the memory state of the periodically-forced ReRAM cell, with *P* set to 3, 4, and 5, respectively.

#### Remark 4

The theoretical framework, presented in this manuscript, provides evidence for the support, which nonlinear system theory may provide to experimenters and circuit design engineers. The experimental validation of the theory is the aim of our future research efforts. There are several challenges to tackle in order to achieve this goal.

1. Memristor devices available today can have limited endurance and their electrical behaviour may be subject to subtle drifts under operation, requiring much care and numerous repetitions to acquire convincing results.
2. Intrinsic variability in memristors requires the procurement of significant statistics, regarding device-to-device and cycle-to-cycle variability effects, for the provision of convincing experimental results.
3. The input pulse sequences, required in our programming schemes, are rather complex, requiring finely-programmable high-frequency pulse generators to support the experimental validation activities. This calls for the need to adapt existing measurement routines, available in house, or to acquire new experimental setups.

In regard to the third challenge from the above list, it might finally turn out to be less problematic than it seems, as explained next. The application of the rigorous system-theoretic methodology, presented in this section, to the Strachan model results in the specification of input pulse widths, decreasing at exponential rate with increases in their heights. While this issue does not undermine the significance of the theoretical work, which is applicable *mutatis mutandis* to any other memristor model, it originates here as the Strachan mathematical description^4^ was not optimised for regions of the state-voltage space, where the ReRAM cell undergoes local fading memory effects, supporting multistable oscillatory operating modes. In fact, in these regions—refer to the order of magnitude of the bell peak value in either of plots (f), (h), and (l) of Fig. 4, extracted from the DRM upon assigning the first, second, and third positive value $V_+$ from the set $\{0.6 \, \text{V},0.8 \, \text{V},1.0 \, \text{V}\}$ to the DC voltage *V*—the Strachan model may overestimate the speed of the oxygen vacancies as they move across the longitudinal extension of the nanodevice during a SET resistance switching process. This issue points to the necessity to retune the Strachan model so as to reproduce more accurately the behaviour of the nanodevice in the state-voltage space domain, where it is subject to local fading memory effects. Importantly, research investigations, applying the proposed systematic methodology to a recent reformulation of the Strachan model^21^, which employs ad hoc functions to limit to some extent the maximum admissible velocity, attainable by the ions under positive voltages, and was introduced to resolve some numerical issues, the original DAE set may suffer from, resulted in a dramatic increase in the minimum pulse width by several orders of magnitude relative to the case, where no upper bound was enforced on the rate of change of the memory state, in various scenarios, where the amplitudes assigned to the SET pulses were found to trigger local fading memory effects in the ReRAM cell. This provides proof-of-concept evidence that the application of our theory to a properly-optimised variant of the Strachan model might lead to the specification of widths and heights for the pulses, composing cyclically the train stimulus, which would be programmable in the control settings of existing physical AC voltage waveform generators.

### Compensating for the drift in the resistance of crosspoint devices under power off conditions

**Figure 16:**
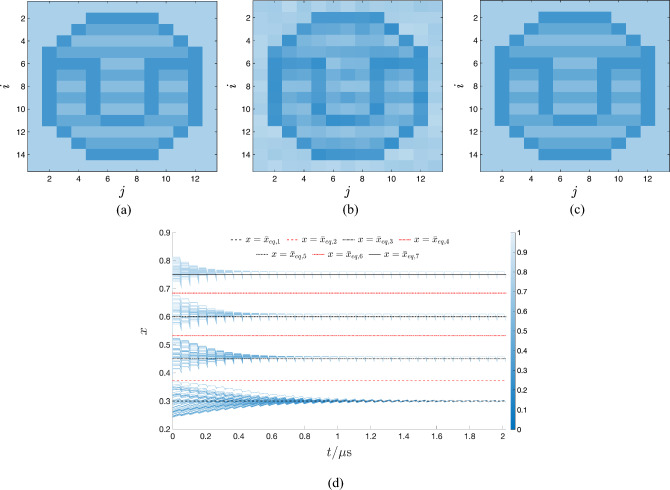
Illustration revealing the possible exploitation of the local history erase effects to compensate for unwanted yet unavoidable drifts in the synaptic conductances of crosspoint nanodevices under power off conditions. (**a**) Initial configuration of the synaptic weight matrix in a nonvolatile memory crossbar with 15 rows and 13 columns. Each of the 195 ReRAM cells, modelled via the Strachan DAE set, is preliminarily programmed in one of four states, specifically $\{0.3,0.45,0.6,0.75\}$, coinciding with the TA-SE equilibria in the case study from Fig. 14. (**b**) Synaptic weight matrix sampled at an arbitrary time instant, during an idle phase, following the programming step, which reveals the detrimental effect of additive noise from a uniform distribution across the range $[-0.06,+0.06]$. (**c**) Retrieval of the original synaptic weight matrix after transients decay to zero during the application of the five-pulse-per-cycle pulse train voltage stimulus $v_S$ from Fig. 14d across each of the 195 crosspoint nanodevices. (**d**) Time course of the states of the crosspoint nanodevices from the respective initial conditions, corresponding to the synaptic weight matrix from (**b**), toward asymptotic oscillations, revolving around their target values. The memory configuration in (**c**) is extracted by sampling simultaneously all the states of the ReRAM cells at the last time instant, corresponding to the end of an input cycle, which is shown along the horizontal axis in (**d**). The colour coding map, depicted in (**d**), applies to all the plots in this figure.

This section presents a potential application, where the multistable oscillatory response of the ReRAM cell to periodic pulse train stimulation may provide benefits for nonvolatile memory crossbar array design. Let us assign the synaptic weights of the 195 ReRAM cells, arranged at regular positions across a nonvolatile memory crossbar array, featuring 15 rows and 13 columns, in such a way that the coloured image, coding them, may form the symbol of TU Dresden, as shown in plot (a) of Fig. 16. This is accomplished by properly programming the state $x_{i,j}$ of the identical ReRAM cell sitting in the position, identified by row index *i* and column index *j*, to either of the four values from the set $\{0.3,0.45,0.6,0.75\}$ ($i \in \{1,2,\ldots ,15\}$, $j \in \{1,2,\ldots ,13\}$). Under power-off conditions, the state of each memristor may drift away from the value, it is supposed to store, due to some unwanted perturbation effect, modelled here as additive noise drawn from a uniform distribution defined over the range $[-0.06,+0.06]$. Suppose that at some time instant after the programming phase, the synaptic weight matrix appears as corrupted as visualised in the illustration from Fig. 16b. Applying the analytical methodology from section 5.3 to endow the TA-SE under $V_-=-0.5\text{V}$ with the 4 stable equilibria $\bar{x}_{\text {eq},1}=0.3$, $\bar{x}_{\text {eq},3}=0.45$, $\bar{x}_{\text {eq},5}=0.6$, and $\bar{x}_{\text {eq},7}=0.75$, corresponding to the levels programmed earlier on into the crosspoint nano-devices of the memory array (recall the exemplary case study illustrated in Fig. 14), the application of the five-pulse-per-cycle pulse train voltage stimulus $v_S$ (refer to plot (d) from that figure) across each of the ReRAM cells allows to recover the original synaptic matrix after transients vanish, as depicted in Fig. 16c. In this example the noise perturbation is not as strong to cause any of the states of the 195 ReRAM cells to move away from the basin of attraction of the oscillation, among the four possible steady-state time waveforms $x_{\text {ss},1}$, $x_{\text {ss},3}$, $x_{\text {ss},5}$, and $x_{\text {ss},7}$ (recall in turn plots (f), (g), (h), and (i) of Fig. 14), revolving around the TA-SE equilibrium, denoting the corresponding original value. Thus, as illustrated in plot (d) of Fig. 16, the state of each of the crosspoint nanodevices progressively approaches the target value, oscillating around it asymptotically.

## Conclusions

Memristor physical realisations are the focus of extensive and intensive research investigations worldwide as their adoption in integrated circuit design may allow to develop innovative technical systems, which exploiting their peculiar capability to combine data sensing, storage, and processing functionalities in a compact nanoscale volume, and leveraging the use of the vertical dimension to accommodate them densely within the oxide-filled gaps, which form at the cross-points between sets of perpendicular and vertically-displaced metal lines, on top of CMOS circuitry, promise to overcome the performance limitations of traditional technical systems, opening a wide spectrum of opportunities for electronics in the post-Moore era. Due to the strong nonlinearity, characterising the operating principles of these nanodevices, recurring to powerful concepts from Nonlinear Circuit and System Theory^2^ is a necessary step for drawing a full picture of their dynamics. In fact the common approach of electrical engineers to linearise the model of a nonlinear device before commencing the investigation of its dynamics is insufficient to explore their global behaviour. As an example of the significant impact that this theory may have on the progress of memristor research, this paper reveals how the application of some of its powerful techniques to a predictive model^4^ of a Ta$_2$O$_{5-\text {x}}$ Resistive Random Access Memory cell from Hewlett Packard Labs may allow the development of a systematic strategy, supported by a rigorous analytical framework, to craft a generalised rectangular pulse train voltage stimulus, composed of $P\in \mathbb {N}_{>0}$ SET positive pulses and of a single RESET negative pulse, so as to endow the memory state of the nano-device with *P* of coexisting oscillatory solutions, revolving around mean values, prescribed as design specification, and observable at steady state for all initial conditions drawn from their basins of attraction. The availability of an algorithm, which, evaluating analytical formulas, and solving a linear system of equations, automatically massages the properties of a generalised pulse train stimulus for triggering a monostable (multistable) periodic response in a Resistive Random Access Memory cell, triggering the emergence of global^3^ (local^9,10^) fading memory effects across its physical medium, and forcing it to oscillate around a specific resistance level, for any initial condition from the state existence domain (from a certain basin of attraction), may inspire the development and circuit implementation of novel in-memory sensing and computing paradigms in the years to come. As an example of a potential application of the theory, the local fading memory effects, emerging in the ReRAM cell according to the Strachan model, have been leveraged to propose a novel scheme to compensate for the unavoidable drift in the resistance of a crosspoint nanodevice under power off conditions. A similar theoretical approach, as the one, presented in this paper for the Strachan model, may be developed to investigate the response of the mathematical description of any other non-volatile^22^ or volatile^23^ resistance switching memory to periodic pulse train stimuli.

## Data Availability

The datasets used and/or analysed during the current study are available from the corresponding author on reasonable request.
