# Author Correction: An analytical approach to engineer multistability in the oscillatory response of a pulse-driven ReRAM

**DOI:** 10.1038/s41598-024-69891-6

**Published:** 2024-09-03

**Authors:** Alon Ascoli, Nicolas Schmitt, Ioannis Messaris, Ahmet Samil Demirkol, John Paul Strachan, Ronald Tetzlaf, Leon Chua

**Affiliations:** 1 Present Address: Technische Universität Dresden, Faculty of Electrical and Computer Engineering, 01069 Dresden, Germany; 2 Technische Universität Dresden, Institute of Circuits and Systems, Faculty of Electrical and Computer Engineering, 01069 Dresden, Germany; 3 RWTH Aachen University, Aachen, Germany; 4 University of California Berkeley, Department of Electrical Engineering and Computer Sciences, Berkeley, CA 94720 USA

Correction to: *Scientific Reports* 10.1038/s41598-024-55255-7, published online 07 March 2024

The original version of this Article omitted an affiliation for Alon Ascoli during the preparation of this research contribution. The correct affiliations are listed below.

Politecnico di Torino, Department of Electronics and Telecommunications, Turin, 10129, Italy

Technische Universität Dresden, Faculty of Electrical and Computer Engineering, Dresden, 01069, Germany

The original Article has been corrected.

